# Improving oxidation stability of 2D MXenes: synthesis, storage media, and conditions

**DOI:** 10.1186/s40580-021-00259-6

**Published:** 2021-03-16

**Authors:** Aamir Iqbal, Junpyo Hong, Tae Yun Ko, Chong Min Koo

**Affiliations:** 1 Materials Architecturing Research Centre, Korea Institute of Science and Technology (KIST), Seoul, 02792 Republic of Korea; 2 Division of Nano & Information Technology, KIST School, University of Science and Technology, Seoul, 02792 Republic of Korea; 3 Department of Materials Science and Engineering, Korea University, Seoul, 02841 Republic of Korea; 4 KU-KIST Graduate School of Converging Science and Technology, Korea University, Seoul, 02841 Republic of Korea

**Keywords:** MXene, Oxidation kinetics, Defect passivation, Organic dispersion, Storage condition, Polymer composite, Chemical etching, Two-dimensional (2D) nanomaterials

## Abstract

Understanding and preventing oxidative degradation of MXene suspensions is essential for fostering fundamental academic studies and facilitating widespread industrial applications. Owing to their outstanding electrical, electrochemical, optoelectronic, and mechanical properties, MXenes, an emerging class of two-dimensional (2D) nanomaterials, show promising state-of-the-art performances in various applications including electromagnetic interference (EMI) shielding, terahertz shielding, electrochemical energy storage, triboelectric nanogenerators, thermal heaters, light-emitting diodes (LEDs), optoelectronics, and sensors. However, MXene synthesis using harsh chemical etching causes many defects or vacancies on the surface of the synthesized MXene flakes. Defective sites are vulnerable to oxidative degradation reactions with water and/or oxygen, which deteriorate the intrinsic properties of MXenes. In this review, we demonstrate the nature of oxidative degradation of MXenes and highlight the recent advancements in controlling the oxidation kinetics of MXenes with several promising strategic approaches, including careful control of the quality of the parent MAX phase, chemical etching conditions, defect passivation, dispersion medium, storage conditions, and polymer composites.

## Introduction

Two-dimensional (2D) MXenes with a general structure of M_*n*+1_X_*n*_T_*x*_, where n = 1–4, are the derivatives of their corresponding three-dimensional (3D) bulk layered materials called MAX (or M_*n*+1_AX_*n*_) phases. After chemical etching of A atomic layers from MAX, these compacted 3D structures yield thin 2D nanosheets of MXenes, retaining the hexagonal crystallinity. The term MXene is used because their morphology is analogous to that of the other 2D materials such as graphene, silicene, and phosphorene [1–3]. In the general structure formula M_*n*+1_X_*n*_T_*x*_, the terms M, A, X, and T_*x*_ stand for early transition metals (Ti, Mo, Nb, V, Zr, Sc, Hf, W), group 13 or 14 elements (Al, Si, Ga), carbon and/or nitrogen, and surface terminations, respectively. Out of over 100 known MAX phases, almost 30 MXenes have been experimentally synthesized and many more are theoretically predicted to exist [3, 4].

Naguib et al. [1] reported the first MXene (Ti_3_C_2_T_*x*_) in 2011 by etching Al-based Ti_3_AlC_2_ MAX in a strong hydrofluoric (HF) acid. The wet etching environment removed the aluminum layers and caused the surface of the MXene sheets to be terminated with functional groups such as =O, –OH, and –F by incorporating flakes with good hydrophilicity and dispersion stability in aqueous dispersions. Unlike other 2D materials, Ti_3_C_2_T_*x*_ MXene, which is the representative of the MXene family, exhibits outstanding electrical, electrochemical, optoelectronic, and mechanical properties and has prompted the exploration of other MXenes with different elemental compositions, namely: M_5_X_4_, M_4_X_3_, M_3_X_2_, and M_2_X. Subsequently, binary transition metal compositions (M_1_ and M_2_) and solid solutions (X_1_ and X_2_) further expanded the list of conventional single M and X layer MXenes, offering a wide range of controllable electronic and optical properties [5–8].

MXenes have attracted significant attention owing to their versatile intrinsic properties, such as an excellent metallic conductivity that originates from excess electron density at the Fermi level (*E*_*F*_) [9], hydrophilicity due to abundant water-loving polar surface terminations [10], and easy solution processability without any need of dispersing agents [11]. These unique properties render them favorable in various potential applications such as electromagnetic interference (EMI) shielding [12–15], terahertz shielding [16, 17], electrochemical energy storage [18–20], optoelectronics [21, 22], flexible and transparent electrodes [23, 24], sensors [25], thermal heaters [26], light-emitting diodes (LEDs) [27, 28], and antibacterial films [29].

However, MXenes suffer from severe oxidative degradation, which drastically deteriorates all their characteristics and hampers further applications. In this regard, improving the oxidation stability of MXenes is a challenging task, to prolong their shelf life for practical applications. In the timespan of a decade (from discovery to 10 years later, 2011–2020), significant improvements in the quality-control and oxidation stability of MXenes have been made, suggesting that each step from synthesis to storage and usage has a strong impact on the resulting features of MXenes. In this review, we aim to improve our understanding of the oxidative degradation of MXenes and summarize all the comprehensive studies that have been conducted to prevent or delay the oxidation kinetics of MXenes. This report elucidates all the possible methods used to improve the oxidation stability of MXenes, thereby providing insights for their practical applications under different conditions.

## Oxidative degradation of MXenes and influencing parameters

**Fig. 1:**
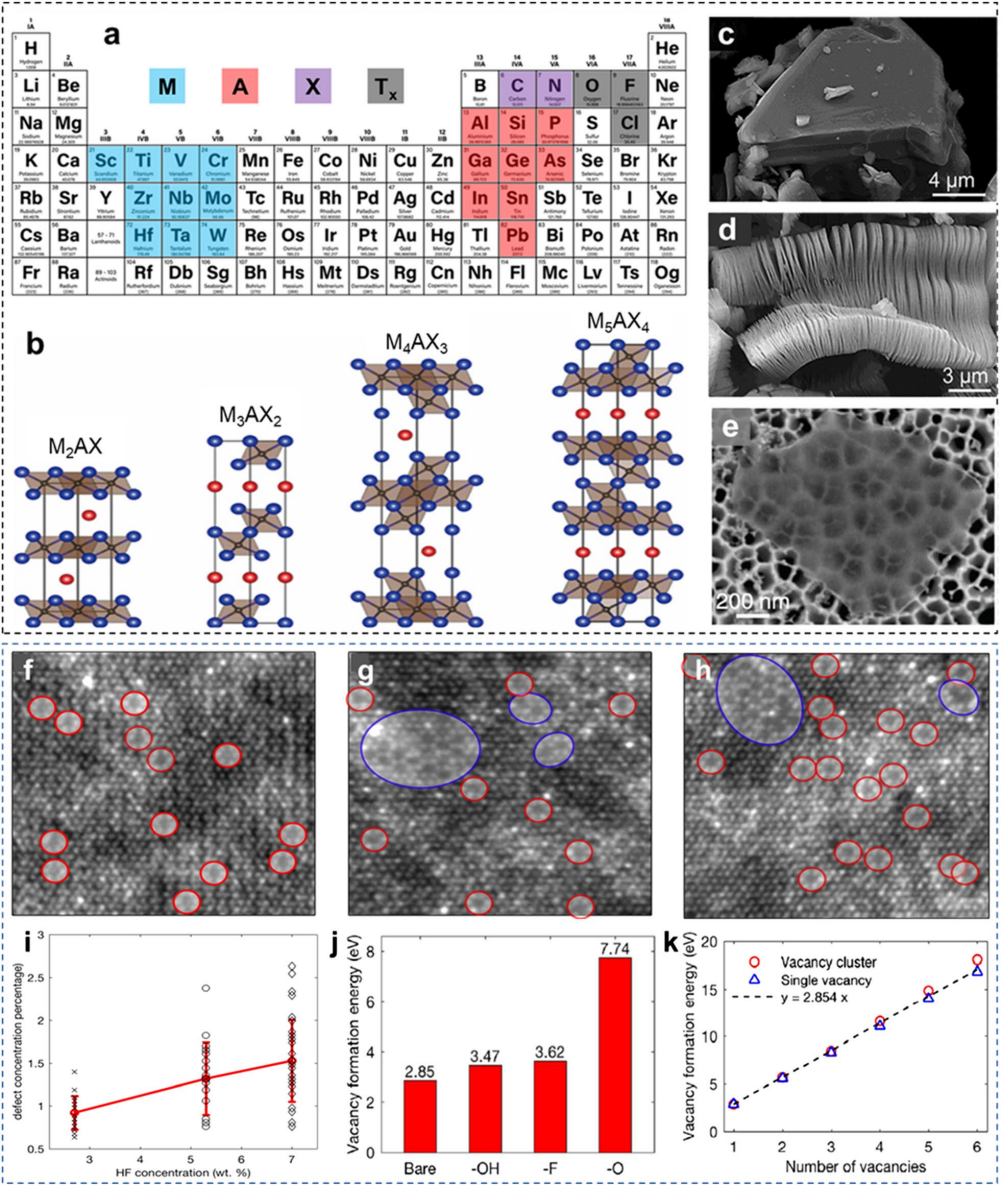
**a** Elements in MAX phases and MXenes. **b** Atomic structures of different MAX phases. Scanning electron microscopy (SEM) images of **c** a Ti_3_AlC_2_ particle and **d** accordion-like multilayer structure of Ti_3_C_2_T_*x*_ MXene after removal of the Al atoms from Ti_3_AlC_2_ by HF etching. **e** Exfoliated single-layer Ti_3_C_2_T_*x*_ MXene sheet on an anodized aluminum oxide disk. HAADF-STEM images from single-layer Ti_3_C_2_T_*x*_ MXene flakes prepared using: **f** 2.7 wt% HF, **g** 5.3 wt % HF, and **h** 7 wt % HF. **i** Scatter plot of defect concentrations from images acquired from samples produced using different HF acid concentrations. The red line shows the error plot with the average and standard deviation for different HF acid concentrations. **j** V_Ti_ formation energy on bare Ti_3_C_2_ and terminated single-layer Ti_3_C_2_T_*x*_. **k** Formation energy of ${V}_{Ti}^{C}$ clusters as a function of number of V_Ti_. **b** Reproduced with permissions from ref. [7]. Copyright (2020), Elsevier. **c**, **d** Reproduced with permissions from ref. [8], Copyright (2012), American Chemical Society. **e** Reproduced with permissions from ref. [12]. Copyright (2016), American Association for the advancement of Science, Science. **f**–**k** Reproduced with permissions from ref. [32]. Copyright (2016). American Chemical Society

MXene is an emerging class of 2D materials [30], which is chemically synthesized from layered MAX phases immersed into acidic solutions (Fig. 1a). To date, MAX phases with the atomic structures of M_2_AX, M_3_AX_2_, M_4_AX_3_, and M_5_AX_4_ (Fig. 1b) have been transformed into their corresponding M_2_X, M_3_X_2_, M_4_X_3_, and M_5_X_4_ MXenes via the same wet chemical synthesis process. The strong M–X bonds in MAX phases allow the selective etching of M–A bonds, resulting in accordion-like multilayer and few-layer MXene structures. Figure 1c–e correspond to the Ti_3_AlC_2_ MAX phase before etching, and the multilayer and monolayer Ti_3_C_2_T_*x*_ MXene after etching, respectively. With the addition of different M and X elemental compositions, synthesized MXenes possess electrical conductivity ranging from 1 S cm^−1^ to as high as ~ 20,000 S cm^−1^ under controlled synthesis conditions [24, 31]. The intrinsic properties of MXenes, including metal-like electrical conductivity, are determined not only by the type and composition of M and X constituents in MXenes [6], but also by many other factors including defect density [32], flake size [33], surface functional groups of the synthesized MXene flakes, and type of intercalants [9, 34]. These factors directly affect the oxidation kinetics of the synthesized MXenes. Therefore, oxidative degradation of MXenes is controlled not only by synthesis parameters including the quality of the parent MAX phase [31, 35], chemical etching conditions such as the type and concentration of acid etchants [36], and ultrasonication and post-etching treatments [11, 37], but also by storage environments such as storage media [38–40], temperature [41], pH [42], and dispersion concentrations [41–43].

In [1], highly concentrated hydrofluoric (HF) acid was used for the synthesis of Ti_3_C_2_T_*x*_ MXene. Unlike graphene and black phosphorous (BP), mechanical exfoliation of MAX phases is difficult; hence, a strong acid is needed to overcome the metallic bonds between M and A elements [1, 44, 45]. The most reactive Al atoms were selectively etched out by reacting them with the strong HF acid, resulting in the formation of accordion-like multilayer Ti_3_C_2_T_*x*_ MXene. Subsequently, mono-to-few-layer Ti_3_C_2_T_*x*_ MXenes were prepared by sonication or intercalating metallic ions into the multilayered laminates [11, 46–50].

Harsh chemical etching synthesis inevitably results in defective MXene flakes, which are the key sites for oxidation reactions and fuel the degradation of MXenes. Sang et al. [32] observed the atomic defects and their response to the concentrations of HF acid in monolayer Ti_3_C_2_T_*x*_ MXene sheets and showed that HF acid etched some exposed Ti atoms along with Al atoms, resulting in Ti vacancies (V_Ti_) and Ti vacancy clusters (${V}_{Ti}^{C}$), as evidenced by the high-angle annular dark field (HAADF)-scanning transmission electron microscopy (STEM) images (Fig. 1f–h). The Ti vacancies were formed in the upper and lower Ti layers that were completely exposed to HF acid, whereas the inner Ti layer adjacent to the C layer was less defective. Theoretically, these results were supported by the difference in the formation energy of vacancies, where 2.842 eV and 6.485 eV were measured for the outer and inner Ti layers, respectively. Therefore, a strong acid concentration will create more Ti vacancies than those formed by a weak acidic concentration. As shown in Fig. 1f, a lower HF acid concentration of 2.7 wt% only created single atom Ti vacancies. Higher concentrations of HF acid (5.3 and 7 wt%) formed a combination of single-atom vacancies and vacancy clusters as shown in Fig. 1g, h where single V_Ti_ vacancies have been indicated as red circles, while vacancy clusters are shown as blue circles. Figure 1i summarizes the defect concentrations measured for tens of flakes for a specific concentration of HF acid, showing a direct relationship between the two parameters. The study also calculated the formation energy of the monolayer Ti_3_C_2_T_*x*_ MXene functionalized with different terminations. In Fig. 1j, –OH terminated MXene showed minimum energy formation of the vacancy, which was extremely similar to that of pristine MXene without any terminal groups. This suggests easy vacancy formation in –OH-terminated MXenes, which also supports the faster oxidation of aqueous MXene dispersions. In contrast, =O terminations render Ti_3_C_2_T_*x*_ MXene highly stable, owing to the higher energy formation required to create Ti vacancies. Moreover, the per-atom formation energy decreased in the case of clusters, indicating the favorable formation of larger vacancy clusters than that of the single-atom vacancies (Fig. 1k). This study highlights that atomic defects in nanometer-thick MXene layers are highly active sites for oxidation, which is prompted by an established intrinsic electric field.

**Fig. 2:**
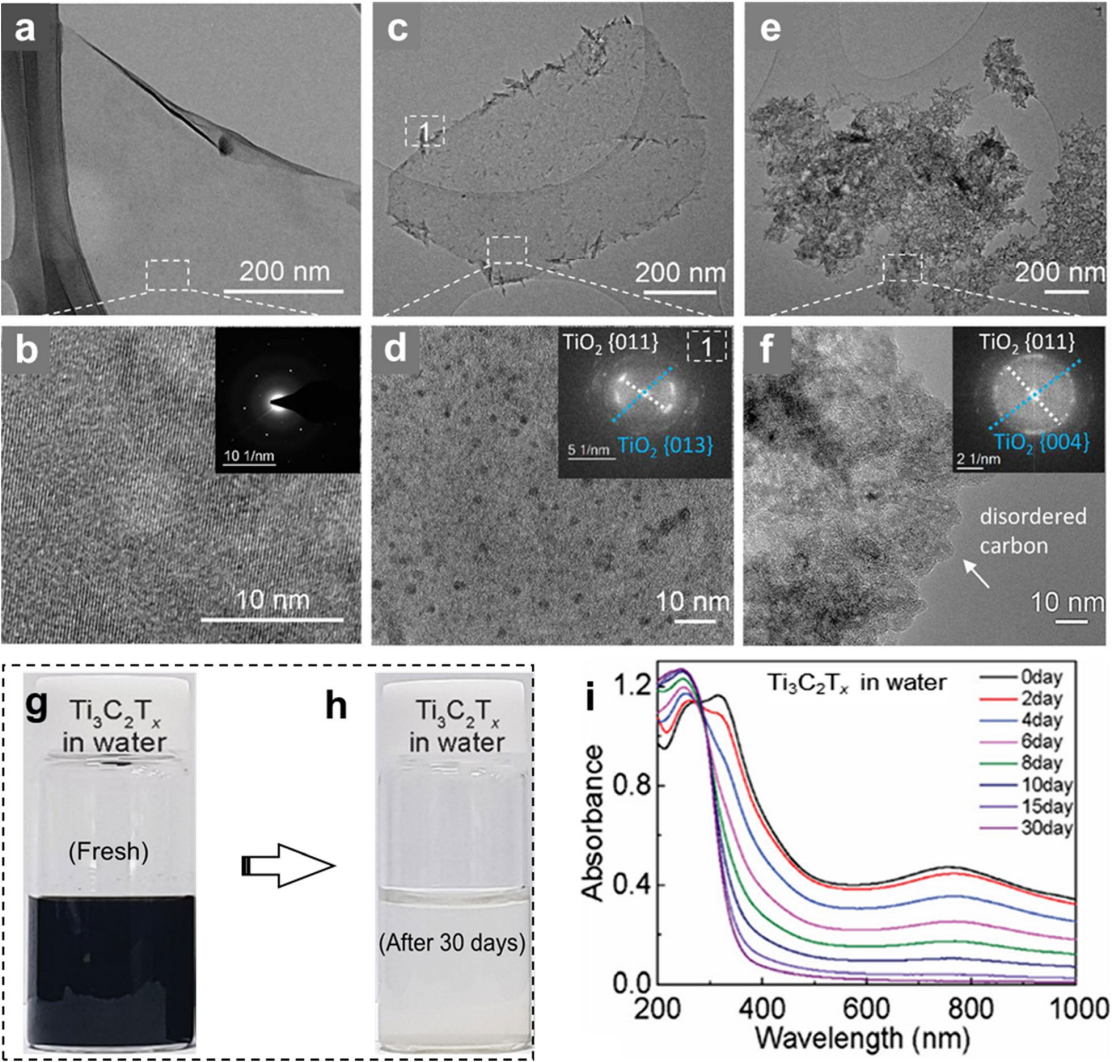
TEM images of **a** MXene flakes from fresh d-Ti_3_C_2_T_*x*_ solution and aged solutions in open air for **c** 7 days and **e** 30 days, respectively. **b**, **d**, **f** The high-resolution TEM images of **a**, **c**, **e**; respectively. Inset in **b** is the corresponding Selected Area Electron Diffraction (SAED) pattern, and those in **d**, **f** are the corresponding FFT patterns. **g**, **h** Photographs of fresh Ti_3_C_2_T_*x*_ MXene in water, and the aged Ti_3_C_2_T_*x*_ MXene dispersion in water after 30 days in open air, showing an opaque whitish color due to oxidation. **i** UV–visible absorbance spectra of pristine Ti_3_C_2_T_*x*_ in water collected over a period of one month. **a**–**f** Reproduced with permissions from ref. [41]. Copyright (2017), American Chemical Society. **g**–**i** Reproduced with permissions from ref. [40]. Copyright (2019), American Chemical Society

Atomic defects are nucleation sites for the oxidation of MXenes. These atomic defects are inevitable in chemical synthesis and hence result in the degradation of MXenes over a period of time. Zhang et al. [41] observed that the synthesized fresh MXene flakes showed very clean edges with a neat surface (Fig. 2a, b). The aging of aerated aqueous MXene dispersions initiated the oxidation of MXene flakes, starting from the edges, where TiO_2_ crystals were observed in the form of branches (Fig. 2c). Figure 2d shows the high-resolution transmission electron microscopy (TEM) image of the MXene flakes where oxidation can be clearly observed. The fast Fourier transform (FFT) image in the inset of Fig. 2d confirms that the branch-like species growing at the edges are TiO_2_ crystals. At this stage, the MXene flakes retained their 2D morphology, implying partial oxidation reactions. However, further aging of the MXene dispersion causes the agglomeration of C atoms, resulting in amorphous carbon and TiO_2_-rich areas, which is indicative of a fully oxidized MXene dispersion (Fig. 2e, f). At this stage, the sheet morphology of MXene flakes was converted into agglomerated TiO_2_ nanocrystals. After complete oxidation, the greenish black dispersion of fresh Ti_3_C_2_T_*x*_ MXene became a cloudy whitish color, owing to the formation of TiO_2_ nanocrystals (Fig. 2g, h).

The UV–vis absorption spectrum is widely used to analyze the degree of oxidation in MXenes because each MXene has its own characteristic absorption peaks at a certain wavelength [11]. Kim et al. [40] investigated the UV–vis spectra of the Ti_3_C_2_T_*x*_ MXene dispersion in water that was stored for one month (Fig. 2i). Fresh Ti_3_C_2_T_*x*_ MXene (greenish black color in Fig. 2g) showed two characteristic peaks at wavelengths of 320 and 760 nm. The intensity of the second peak (at 760 nm) gradually decreased with increasing storage time and completely diminished after 15 days. This indicated the gradual progression of oxidation of MXene dispersion, which was confirmed by an emerging peak of TiO_2_ at around 250 nm. Other studies have also reported a time period of two weeks for the complete oxidation of the Ti_3_C_2_T_*x*_ MXene dispersion under ambient conditions [41, 51]. Using the intensity of peaks in the UV–vis spectra, the stability limit of MXene dispersion at a certain time (t) can be calculated in the form of its decay constant (days) (Eq. 1).

1 $$A={A}_{unre}{+{ A}_{re}\times e}^{(-t/\uptau )}$$

where A, A_unre_, and A_re_ represent the normalized absorbance of all MXene flakes, unreacted/stable flakes, and reactive/unstable flakes at a certain wavelength, respectively; τ is the decay constant (days) at a certain period of time (*t*). The steady decrease in the intensity of the peak at 760 nm is a reliable graphical representation of the degree of oxidation of MXenes.

**Fig. 3:**
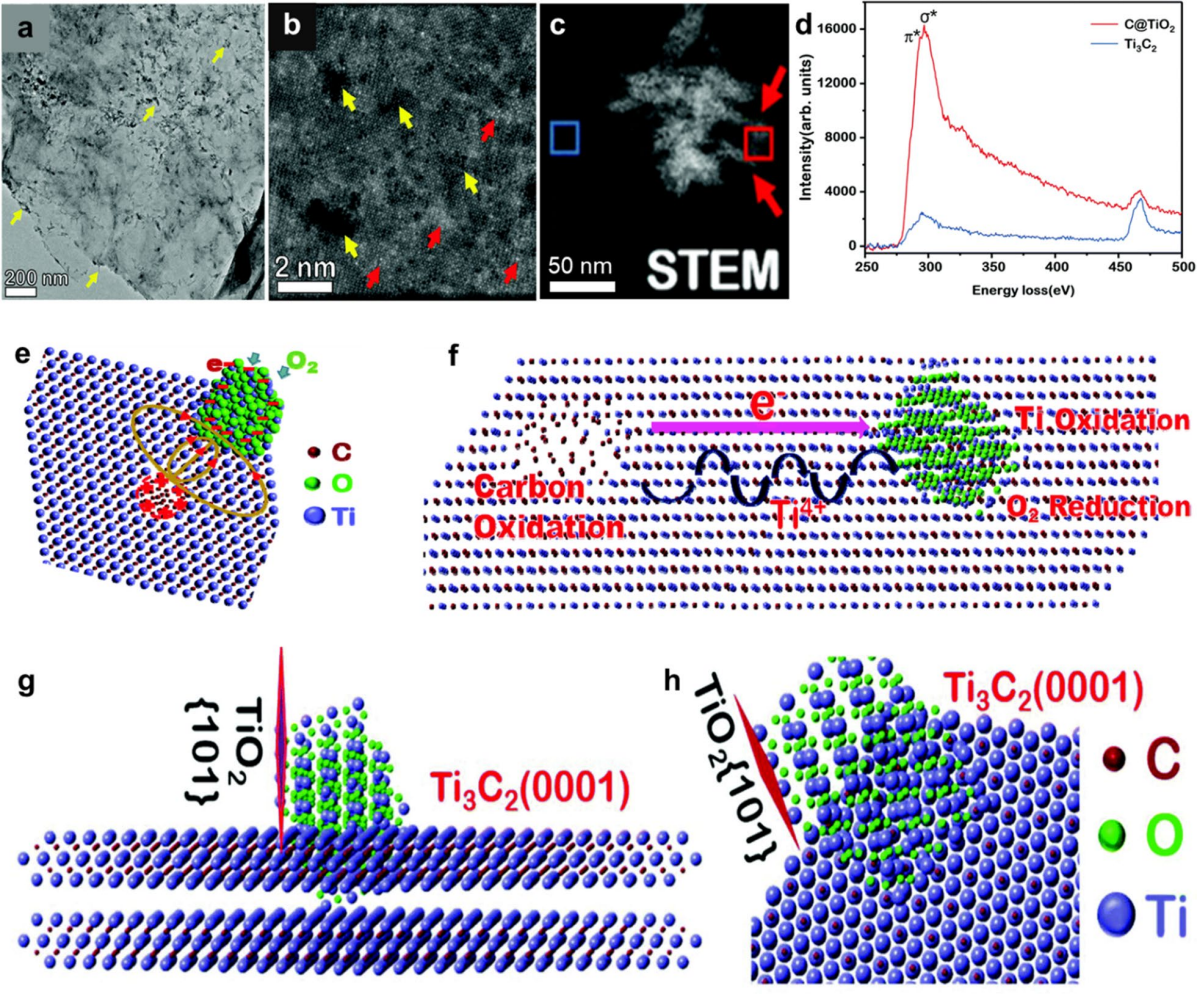
**a** Bright-field TEM image of Ti_3_C_2_ MXene flakes showing the formation of TiO_2_ nanoparticles on edges as well as on the basal plane. **b** A Z-contrast STEM image showing the Ti-vacancies (dark contrast marked by yellow arrowheads) and Ti–rich areas (bright contrast marked by red arrowheads). **c** HAADF-STEM image and elemental maps of C, O, and Ti collected by EELS, showing the coexistence of TiO_2_ nanoparticles and amorphous carbon aggregation. **d** A comparison of EELS spectra, collected from the carbon aggregation marked by the red box in **c** and the Ti_3_C_2_ substrate marked by the blue box. **e** Schematic of the internal electric field, with the positive side formed around a hole with many Ti-vacancies and the negative side with the accumulation of electrons on the convex particle. **f** Illustration of Ti_3_C_2_ oxidation with carbon being oxidized on the positive side of the internal electric field and Ti-ions being oxidized on the negative side. **g**, **h** Illustration of the TiO_2_ cluster on Ti_3_C_2_ with the TiO_2_-(101) plane perpendicular to the MXene basal plane (0001). **a**–**h** are reproduced with permissions from ref. [52]. Copyright (2019), Royal Society of Chemistry

Xia et al. [52] reported the oxidation of Ti_3_C_2_T_*x*_ MXene under ambient conditions (room temperature), where dispersions of synthesized MXenes were stored in tin (Sn)-wrapped vials to neglect the probability of light-induced oxidation. Further, the mechanism behind the oxidation of MXene (Ti-based, Ti_3_C_2_T_*x*_) was explored. In the aged (5 days old) MXene samples, TEM images showed that the oxidation of MXene (formation of TiO_2_ nanocrystals) started from the neighboring atomic defects at the edges and then propagated to the basal plane of the MXene sheets (Fig. 3a). The high-resolution STEM image in Fig. 3b shows the atomic defects marked with yellow arrowheads, whereas the bright spots are assigned to Ti–rich areas, governed by the higher atomic number of Ti atoms. Ti atoms in the MXene layers are oxidized to form TiO_2_ nanoparticles, whereas C atoms in the MXene layers agglomerate and develop an amorphous carbon structure. The formation of TiO_2_ nanocrystals and amorphous carbon is explained by the following equation [52]:

2 $${\text{Ti}}_{{3}} {\text{C}}_{{2}} + {\text{ 3O}}_{{2}} = {\text{ 3 TiO}}_{{2}} + {\text{ 2C}}$$

The HAADF-STEM image in Fig. 3c shows distinct amorphous carbon and TiO_2_ nanoparticles in red and blue marked rectangles, respectively. Electron energy loss spectroscopy (EELS) (Fig. 3d) confirmed the presence of a C–C π* edge at 280 eV taken from the red rectangular area. The EELS spectra collected from the blue rectangular area do not show any C–C π* bonding, validating the unoxidized Ti_3_C_2_T_*x*_ MXene region. Atomic defects are inevitably produced on MXene flakes due to wet chemical synthesis. These atomic defects are the nucleation sites for oxidation reactions, where the oxidation of Ti atoms creates Ti cations in the vicinity of the atomic defects and C atoms form amorphous carbon through oxidation at the Ti vacancies. Owing to the creation of the Ti vacancies, the flow of electrons creates an internal electric field. As Ti vacancies act as positively charged regions, the neighboring C^4−^ ions lose electrons and oxidize, thereby promoting the oxidation reaction. With the growth of TiO_2_ nanoparticles, the number and size of the holes increase gradually, indicating the effects of the initiated Ti vacancy on further oxidation of Ti_3_C_2_T_*x*_ MXene. For carbon oxidation, the accumulated electron holes create excess positive charges, whereas atomic defects create negative charges owing to excess electrons (Fig. 3e). These positive and negative ends create an internal electric field over a distance of a few nanometers and enable Ti cations to move on the surface. The accumulation of radical electrons allows O_2_ (in the form of O^2−^) to enter the lattice. This makes Ti cations diffuse toward the negative side of the electric field, thereby forming TiO_2_ and leading to the oxidation of C atoms on the positive side. This built-up internal electric field causes the diffusion of Ti ions, where the transportation of ions and electrons prompts the nucleation and growth of TiO_2_ (Fig. 3f). The growth direction of anatase TiO_2_ nanocrystals was preferentially favorable in the (101) lattice plane owing to its lowest average surface energy of 0.44 J m^−2^ as compared to 0.90 and 0.53 J m^−2^ for the (001) and (100) planes, respectively. Figure 3g and h show that the (101) plane of the TiO_2_ growth is perpendicular to the basal plane (0001) of the Ti_3_C_2_T_*x*_ MXene flakes. These oxidation reactions in Ti_3_C_2_T_*x*_ MXene are thermodynamically stable, implying that oxidation will continue once it starts until complete degradation of the structure.

Huang et al. [38] proposed that hydrolysis is the main chemical reaction for Ti-based MXenes, whereas direct oxidation by oxygen is less likely to occur. Carbon atoms most likely form CO_2_, carbonic acid (H_2_CO_3_), or other gaseous compounds and hydrocarbons such as methane gas. The hypothetical chemical reactions with different functionalities of the Ti-based MXenes are represented as follows:

3 $${\text{2Ti}}_{{3}} {\text{C}}_{{2}} {\text{O}}_{{2}} + {\text{ 11 H}}_{{2}} {\text{O }} = {\text{ 6TiO}}_{{2}} + {\text{ CO }} + {\text{ CO}}_{{2}} + {\text{ 2CH}}_{{4}} + {\text{ 7H}}_{{2}}$$

or

4 $${\text{2Ti}}_{{3}} {\text{C}}_{{2}} \left( {{\text{OH}}} \right)_{{2}} + {\text{ 11 H}}_{{2}} {\text{O }} = {\text{ 6TiO}}_{{2}} + {\text{ CO }} + {\text{ CO}}_{{2}} + {\text{ 2CH}}_{{4}} + {\text{ 9H}}_{{2}}$$

The formation of CO_2_ and H_2_CO_3_ was confirmed by the decreased pH values (shifting toward acidic values) of the aqueous dispersions of Ti_2_CT_*x*_ MXene. However, the non-proportional relationship between the decreased pH value and increased atomic percentage of Ti(IV) does not prove this hypothesis [42]. Huang and co-worker later claimed that only CH_4_ is released during degradation of carbide MXenes, whereas carbonitride MXene (Ti_3_CNT_*x*_) released NH_3_ as well [53]. After considering the oxidation mechanism, we summarize possible ways to minimize or delay the oxidation degradation of MXenes under different controlled conditions.

## Methods for improving the oxidation stability of MXenes

### Synthesis of minimal defective layered MXenes

Every step in the synthesis of MXenes affects their final structure, physiochemical properties, and the oxidation stability [54]. MXenes are topochemically derived from their parent MAX phases; therefore, the structure and properties of MXenes are highly affected by the quality of the MAX phase. The quality of the MAX phase is affected by the choice of the precursors. Raw materials of the MAX phase directly define the properties of the ensuing MXenes [35]. For example, Ti_3_AlC_2_ MAX synthesized from a graphite-based carbon precursor produces more conductive (4400 S cm^−1^) Ti_3_C_2_T_*x*_ MXene with good stability (time constant of 10.1 days). However, MAX synthesized from the TiC-based carbon precursor produces comparatively less conductive (3480 S cm^−1^) and least stable (4.8 days) Ti_3_C_2_T_*x*_ MXene. Similarly, MAX with the lampblack carbon source results in the least conductive (~ 1020 S cm^−1^) and less stable (5.1 days) Ti_3_C_2_T_*x*_ MXene [35]. Note that synthesis of MXenes is quite challenging; however, one can obtain MXenes with the required properties by carefully controlling all the synthesis parameters.

The first Ti_3_C_2_T_*x*_ MXene was synthesized by chemical etching of Ti_3_AlC_2_ MAX in a strong HF acid [1]. After HF etching, several HF-containing and HF-forming methods have been reported for the synthesis of Ti_3_C_2_T_*x*_ and other MXenes [11, 55, 56]. Significant progress was made by introducing less harsh etching conditions for the delamination of Ti_3_AlC_2_ MAX to Ti_3_C_2_T_*x*_ MXene, where a mixture of lithium fluoride (LiF) and hydrochloric (HCl) acid formed in-situ HF acid, resulting in minimal intensive layer delamination (MILD) for the synthesis of Ti_3_C_2_T_*x*_ MXene [55]. This method produced clay-like few-layer MXenes due to high yields. Moreover, less-defective MXene nanosheets showed higher electrical conductivity, which was further improved by tuning the molar ratio of LiF and HCl salts [12]. This etching method has been widely used for a number of applications because it requires no sonication or intercalation-type post-etching treatment [11].

**Fig. 4:**
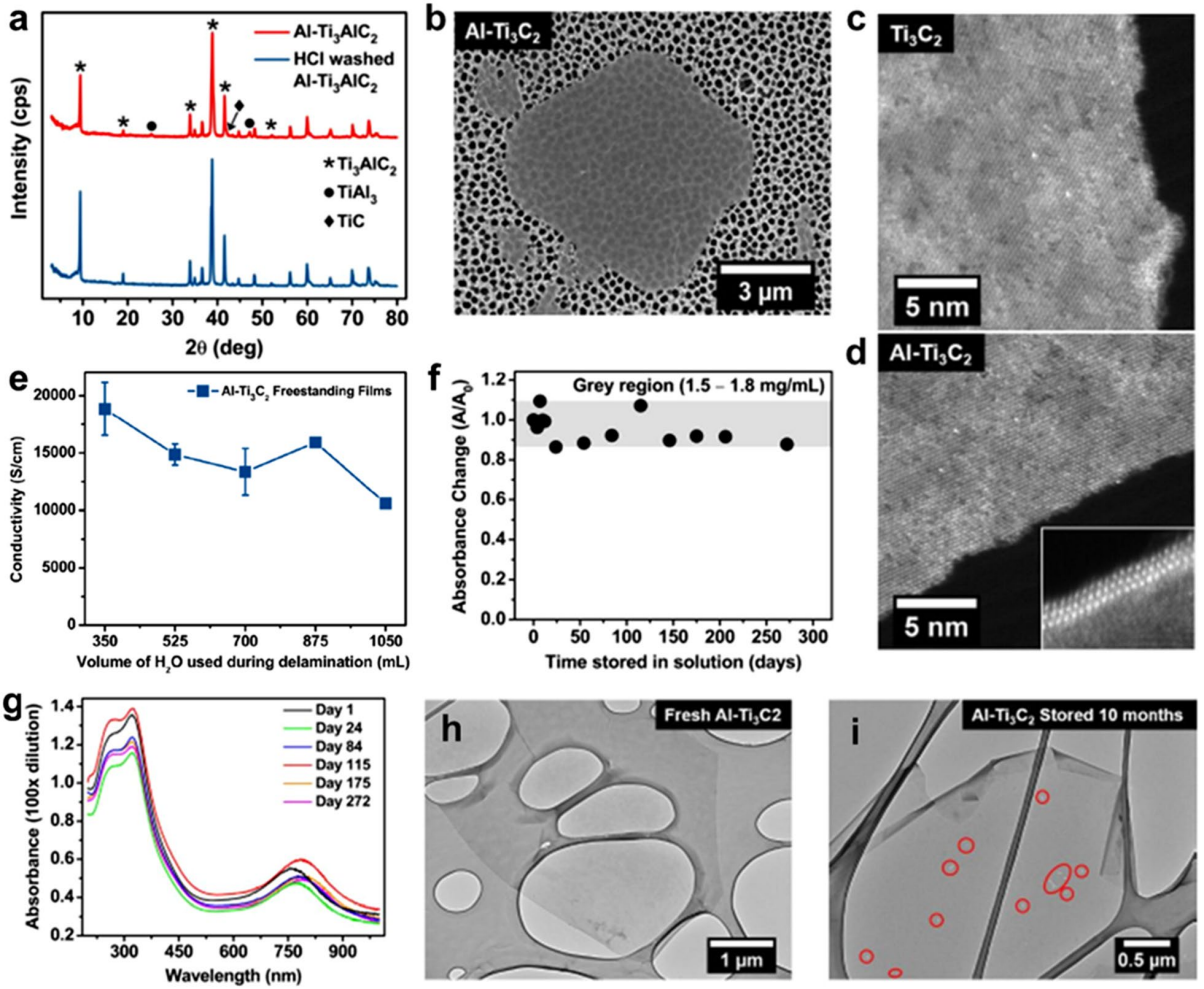
**a** X-ray diffraction patterns of Al-Ti_3_AlC_2_ before (red) and after (blue) HCl washing. **b** SEM image of a hexagonal, single-layer flake of Al-Ti_3_C_2_ produced via HF/HCl etching and LiCl delamination. **c**, **d** High-angle annular dark-field scanning transmission electron microscopy (STEM) images of Ti_3_C_2_ flake edges produced from conventional Ti_3_AlC_2_, and Al-Ti_3_AlC_2_, respectively. **e** Electronic conductivity of freestanding films produced by vacuum-assisted filtration of Al-Ti_3_C_2_ suspensions at different stages of the delamination process. **f** Absorbance changes over time for the stored Al-Ti_3_C_2_ solution calculated from the UV–vis spectra in **g**. The grey region corresponds to the suspension concentrations of 1.5–1.8 mg/mL. **g** UV–vis spectra recorded over time for an aqueous Al-Ti_3_C_2_ solution stored in ambient conditions. The TEM images of a fresh Al-Ti_3_C_2_ flake (h) and an Al-Ti_3_C_2_ flake from a ten-month-old solution (i). The red circles mark all the observable pinholes in the flake. **a**–**i** Reproduced with permissions from ref. [31]

Pure MAX phase synthesized in stoichiometric amounts typically results in high-quality MXenes after etching. However, Mathis et al. [31] synthesized Ti_3_AlC_2_ MAX with excess Al powders during the pressureless sintering process and named it Al-Ti_3_AlC_2_. The synthesized Al-Ti_3_AlC_2_ MAX had traces of TiAl_3_-like intermetallic compounds, which were subsequently removed by washing with HCl acid at room temperature, as shown in the X-ray diffraction (XRD) patterns in Fig. 4a. It is highly important to remove excess aluminum traces because they have a negative impact on the properties of the formed MXene. This HCl-washed Al-Ti_3_AlC_2_ MAX was dried under vacuum, followed by etching in a mixture of HF/HCl and delamination in the aqueous LiCl solution. Figure 4b shows the scanning electron microscopy (SEM) image of the obtained Al-Ti_3_C_2_ MXene flake with lateral dimensions of few microns. The HR-STEM images of the edges taken from the Ti_3_C_2_ MXene (synthesized with conventional Ti_3_AlC_2_ MAX) and Al-Ti_3_C_2_ MXene flakes in Fig. 4c and d, respectively, indicate very clean edges for the latter and distorted edges for the former. As we know that defective edges are the most active sites for the start of oxidation, Al-Ti_3_C_2_ MXene is expected to show improved oxidation stability. In the synthesis of Al-Ti_3_C_2_ MXene, every step was carefully considered. The amount of water used in the washing process was quite effective in defining the quality of MXene, as confirmed by the calculated electrical conductivity value. In the case of the lowest water content (350 mL), the highest electrical conductivity of 20,000 S cm^−1^ was achieved (Fig. 4e); however, minor traces of LiCl salt remained in the MXene dispersion. Higher water content helped remove the LiCl intercalant but at the expense of electrical conductivity. However, this method reported the highest electrical conductivity measured to date. Because of the quality-controlled synthesis process, the concentration of the obtained MXene dispersion was marginally changed, as shown by the periodic absorption change in the UV–vis measurements (Fig. 4f). In addition, no change in the intensity of peaks in the UV–vis spectra reinforced the quality of MXene that was retained well over a period of almost four months (Fig. 4g), whereas prolonged exposure resulted in a red shift of the peak intensity, thereby indicating the initiation of oxidation. This indication was further analyzed by comparing the TEM images of freshly synthesized Al-Ti_3_C_2_ MXene and the 10-month aged Al-Ti_3_C_2_ MXene (Fig. 4h, i). The flakes of Al-Ti_3_C_2_ were quite stable and retained sharp edges as well; however, some pinholes were observed on the lateral surface of the Al-Ti_3_C_2_ MXene flakes. This MAX-controlled synthesis of Al-Ti_3_C_2_ MXene will help expand the real applications of MXenes, owing to their highly improved oxidation stability.

**Fig. 5:**
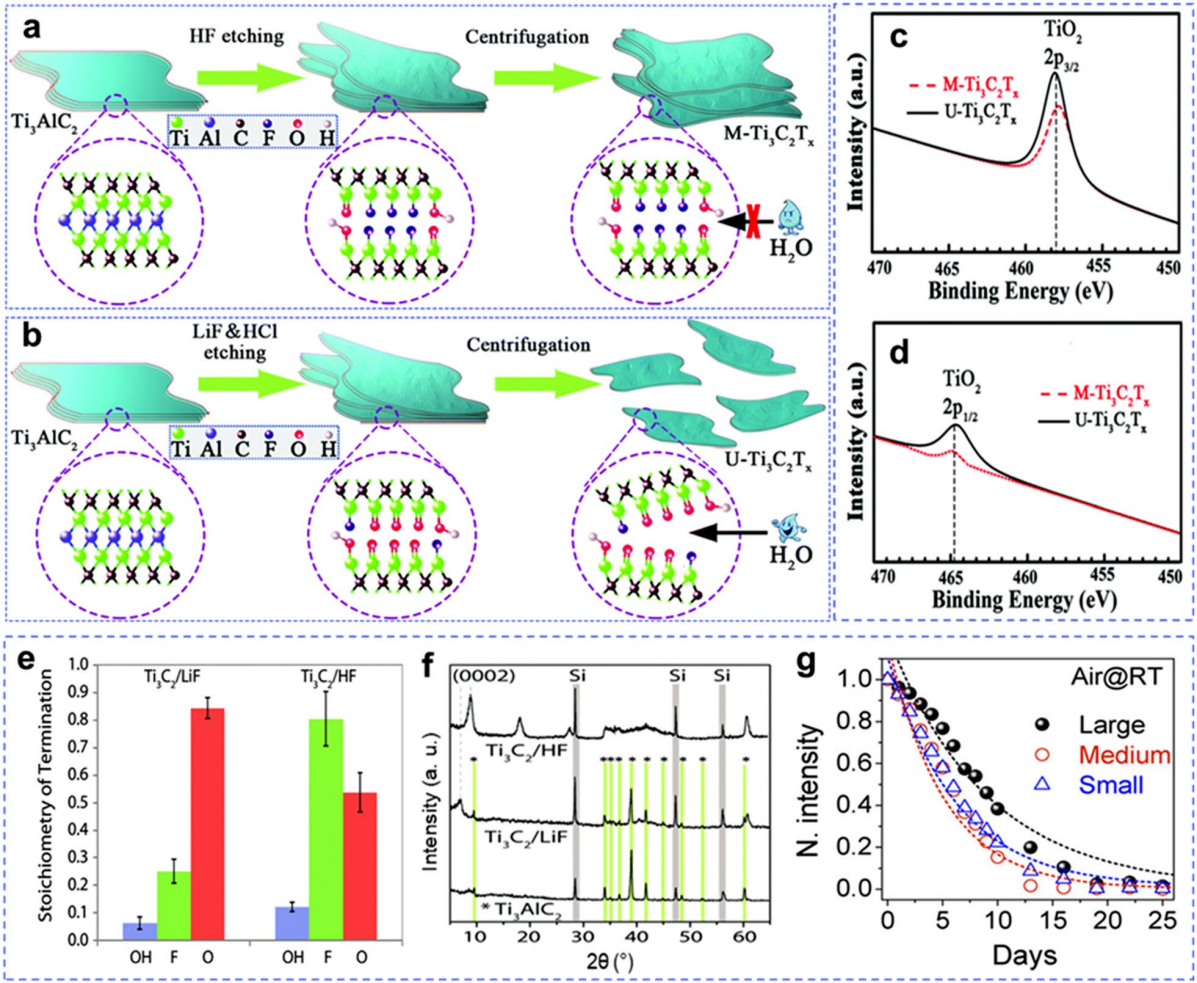
Synthetic illustration of **a** M-Ti_3_C_2_T_*x*_, and **b** U-Ti_3_C_2_T_*x*_ MXenes. Comparison of component peak-fitting of XPS spectra for M-Ti_3_C_2_T_*x*_, and U-Ti_3_C_2_T_*x*_ Ti2p at **c** TiO_2_ 2p_3/2_, and **d** TiO_2_ 2p_1/2_. **e** Composition of the Ti_3_C_2_T_*x*_ surface functional groups produced by etching of the Ti_3_AlC_2_ in HF and LiF–HCl solutions, per Ti_3_C_2_ formula unit, i.e., Ti_3_C_2_(OH)_*x*_F_*y*_O_*z*_. **f** XRD patterns of Ti_3_AlC_2_ and Ti_3_C_2_T_*x*_ etched with HF or LiF–HCl. Reproduced with permissions from the Royal Society of Chemistry. **g** Stability of Ti_3_C_2_T_*x*_ aqueous colloidal solutions with small, medium, and large flake sizes. **a**–**d** Reproduced with permissions from ref. [57]. Copyright (2019), Royal Society of Chemistry. **e**, **f** Reproduced with permissions from ref. [58]. Copyright (2016), Royal Society of Chemistry. **g** is reproduced with permissions from ref. [41]. Copyright (2017), American Chemical Society

After careful selection of the MAX phase, the optimum synthesis conditions directly influenced the properties of the final MXenes. As shown in Fig. 1f–k, the concentration of the acid etchant defines the type and density of atomic defects, which originate the oxidation reactions. A dilute acid solution can help to maintain the integrity of the MXene flakes. He et al. [57] studied the electronic properties and oxidation kinetics of Ti_3_C_2_T_*x*_ MXene, which was synthesized via two different routes: (1) using concentrated (40 wt%) HF at room temperature for 24 h, and (2) using 6 M HCl + LiF at 40 °C for 16 h. The first method produced multilayer MXene (denoted as M-Ti_3_C_2_T_*x*_), whereas the second method produced ultrathin MXene (U-Ti_3_C_2_T_*x*_) sheets (Fig. 5a, b). Between the two synthesized MXenes, M-Ti_3_C_2_T_*x*_ was rich in –F terminations, whereas U-Ti_3_C_2_T_*x*_ exhibited more = O terminations. A similar trend was observed via NMR analysis (Fig. 5e) [58]. Unlike in [32], where the energy for vacancy formation in = O terminated MXene was higher (double) than that of -F-terminated MXene, U-Ti_3_C_2_T_*x*_ showed poorer oxidation resistance than that of M-Ti_3_C_2_T_*x*_, as confirmed by the intensity of the TiO_2_ peaks in the X-ray photoelectron spectroscopy (XPS) spectra (Fig. 5c, d). This result was attributed to the large space available for water molecules to be adsorbed between the MXene layers, thereby increasing the rate of oxidation. Moreover, HF etching fully etched out the Al atoms, whereas minor traces of Al atoms were found in the MXene etched by HCl (Fig. 5f).

The flake size of the synthesized MXene is another parameter that controls the rate of oxidation and degradation [33, 41, 51]. In addition to the concentration of acid etchant [36], ultrasonication is a process that controls the size of MXene flakes. It delaminates the multilayered structures into ultrathin few layers by applying ultrasonic waves that shatter larger flakes into smaller ones, which are collected through centrifugation at a certain revolution per minute (rpm) value [33]. Therefore, the HCl + LiF method provides MXenes with larger flake sizes because no sonication or intercalation is required to delaminate the expanded MXene layers. The flakes of larger sizes are more stable and oxidation-resistant than the smaller ones. A higher exposed surface area of the edges in the smaller flakes favors faster oxidation. UV–vis spectra of Ti_3_C_2_T_*x*_ MXene with different flake sizes show different intensities of the characteristic peak at 760 nm, which is directly related to the size of the flakes. Figure 5g presents the normalized intensity of the 760 nm peak for small, medium, and large flake sizes and shows that the larger flakes are more stable against oxidation than small and medium flakes. This correlation between the type of acid and its concentration encourages the selection of a proper synthesis method to obtain high-quality MXenes with higher oxidation resistance.

### Storage conditions for aqueous dispersion

**Fig. 6:**
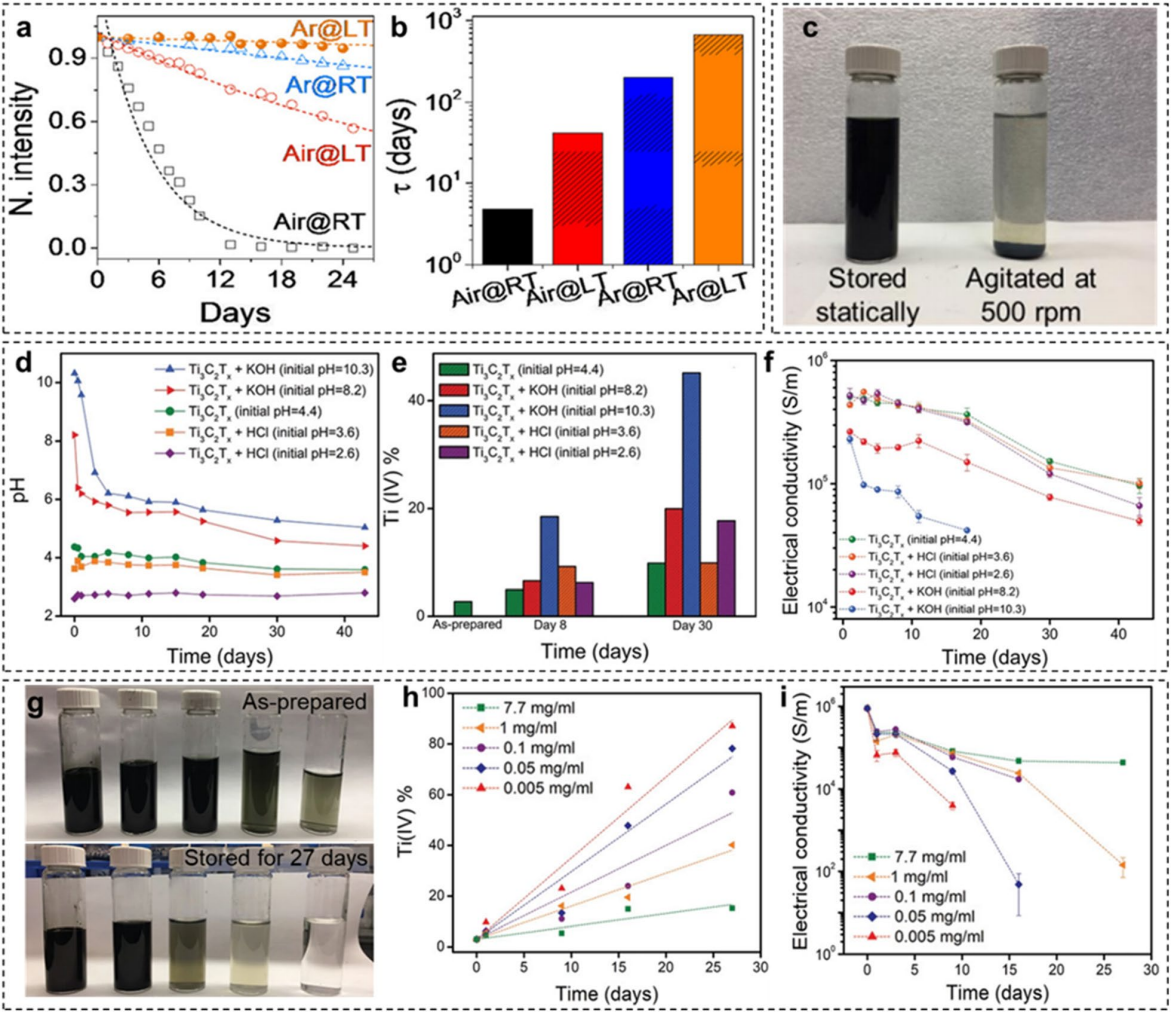
**a** Stability of colloidal d-Ti_3_C_2_T_*x*_ in different environments. **b** Time constants of colloidal d-Ti_3_C_2_T_*x*_ in different environments. **c** Photograph of Ti_3_C_2_T_*x*_ MXene dispersion stored statically (left) and by continuous stirring (right) after 20 days. **d** pH of Ti_3_C_2_T_*x*_ MXene aqueous dispersion as a function of time. **e** Atomic percentage of Ti(IV) obtained by X‐ray photoelectron spectroscopy for Ti_3_C_2_T_*x*_. **f** Electrical conductivity of vacuum‐filtered films made from aged Ti_3_C_2_T_*x*_ MXene dispersions. **g** Photograph of as‐prepared MXene dispersions and the same dispersions on day 27 (from left to right: 7.7, 1, 0.1, 0.05, 0.005 mg mL^−1^). **h**, **i** Atomic percentage of Ti(IV) and electrical conductivity of Ti_3_C_2_T_*x*_ dispersions at different storage times, respectively. **a**, **b** Reproduced with permissions from ref. [41]. Copyright (2017), American Chemical Society. **c**–**i** Reproduced with permissions from ref. [42]. Copyright (2020), Wiley–VCH

The oxidation kinetics of the aqueous dispersion of MXenes depends on the storage conditions such as the storage environment, temperature, pH, and concentration of colloidal dispersions. Zhang et al. [41] discovered an efficient way to improve the oxidation stability of MXenes by analyzing their degree of oxidation under four different conditions. They proposed that after the controlled synthesis of MXene, a controlled storage environment can help prolong and delay the oxidation reactions. A lower storage temperature can significantly delay the oxidation process, but it is not sufficient. When Ti_3_C_2_T_*x*_ MXene was stored under an inert atmosphere in argon-filled vials, the oxidation stability was significantly improved because oxygen, the main source of oxidation, was absent. Hence, the removal of dissolved oxygen improved the oxidation stability of aqueous MXene dispersions. Furthermore, the synergistic effect of lower temperature and the absence of oxygen (MXenes stored in Ar) dramatically enhanced the oxidation stability of aqueous MXene dispersions (Fig. 6a). The time constant of the aqueous Ti_3_C_2_T_*x*_ MXene dispersion increased from a few days to a couple of months (Fig. 6b). The study applied this strategy to the flake size of Ti_3_C_2_T_*x*_ MXene. As smaller flakes oxidize faster than the larger flakes, their propensity to oxidation was halted in an Ar environment at a low temperature.

Zhao et al. investigated the role of particle–particle interactions in the oxidation kinetics [42]. Ti_3_C_2_T_*x*_ MXene at a fixed concentration (3.6 mg mL^−1^) was magnetically stirred in the dark for 20 days at 500 rpm. For comparison, the same dispersion was statically stored under identical environmental conditions. The dispersion agitated by magnetic stirring was highly oxidized compared to the statically stored dispersion. The accelerated oxidation was attributed to the disrupted steric shielding effect between MXene flakes. Ti_2_CT_*x*_ MXene followed the same trend with faster oxidation kinetics.

The pH of MXene dispersions has a large impact on oxidation kinetics. The authors investigated the effect of initial pH on the oxidation stability of Ti_3_C_2_T_*x*_ MXene dispersions [42]. Initial pH (4.4) of the Ti_3_C_2_T_*x*_ MXene dispersion was adjusted by adding 0.1 M HCl and KOH solutions. The increase in pH with the addition of KOH drastically decreased in the first 2 days, followed by a gradual decline over time (Fig. 6d). The sudden consumption of the introduced hydroxyl ions (OH^–^) resulted in a declining drift in the pH. The OH^–^ ions reacted with the –OH terminations of the MXene surface as follows:

5 $${\text{Ti}}_{{3}} {\text{C}}_{{2}} {\text{OH }} + {\text{OH}}^{-} = {\text{Ti}}_{{3}} {\text{C}}_{{2}} {\text{O}}^{-} + {\text{ H}}_{{2}} {\text{O}}$$

Less stable O^–^ groups made MXene surfaces more reactive, resulting in accelerated oxidation. In addition, electrostatic interactions between OH^–^ ions and positively charged edges of the flakes accelerated the oxidation reaction. Thus, alkaline electrolytes caused the degradation of MXene electrodes used for energy storage applications. Ti_3_C_2_T_*x*_ MXene showed a lower oxidation rate under acidic conditions, similar to that of MXene dispersions without pH adjustment. These reactions were confirmed by quantitative measurements of the atomic fraction of Ti(IV) and the electrical conductivity of the dispersions at certain periods of time (Fig. 6e, f). The increased Ti(IV) content and the decreased electrical conductivity values indicate the degree of oxidation in the dispersions. This indicates that acidic dispersions of MXenes are more environmentally stable. The dispersion of Ti_2_CT_*x*_ MXene followed the same trend with a drastic increase in Ti(IV) content and decreased electrical conductivity, indicating faster oxidation kinetics.

The authors extended their work to investigate the effect of the concentration of MXene dispersion on the rate of oxidation [42]. As shown in Fig. 6g (from left to right), the dispersions of Ti_3_C_2_T_*x*_ MXene with higher concentration are oxidized more slowly than diluted dispersions, owing to the steric shielding effects between the flakes in close proximity. Figures 6h, i are consistent with Fig. 6e, f. A higher concentration of the dispersion does not allow water molecules to enter between the MXene layers, thus hindering the oxidation kinetics. All these studies emphasize the efficient storage of MXene dispersions to decelerate their rate of oxidation.

Habib et al. [51] explained the gradual oxidation of Ti_3_C_2_T_*x*_ MXene dispersions (stored under different conditions) in terms of the electrical conductivity measured on free-standing bucky papers obtained by vacuum-assisted filtration. A sharp decrease in electrical conductivity was observed for the Ti_3_C_2_T_*x*_ MXene dispersions stored in open air; i.e., electrical conductivity of fresh Ti_3_C_2_T_*x*_ MXene (2.49 × 10^4^ ± 1.16 × 10^3^ S m^−1^) decreased by 93% in only 27 days. Moreover, Ti_3_C_2_T_*x*_ MXene aqueous dispersions stored in ice (at 0 °C) had stable electrical conductivity values, indicating a slower oxidation process. A slower oxidation rate was observed in the polymeric composites of Ti_3_C_2_T_*x*_ MXene with polyvinyl alcohol (PVA). This was governed by the hydrogen bonding between MXene nanosheets and polymeric chains, resulting in an MXene/PVA sandwich structure with improved oxidation stability. The authors also demonstrated faster oxidation kinetics of Ti_3_C_2_T_*x*_ MXene under UV light, where a few hours of UV exposure faded the electrical conductivity of MXene by triggering oxidation reactions. This result suggested not to dry MXene samples under a UV lamp, to avoid any misinterpretation of the studies and the results.

### Passivating the defects of MXene flakes in aqueous dispersions

MXenes with excellent water dispersibility are easy to oxidize because of the hydroxyl groups on the MXene surface, which are attacked by dissolved oxygen and water molecules that cause the MXenes to lose their original excellent electrical conductivity. In general, the edge site of MXene is more vulnerable to water molecules and/or dissolved oxygen than the base surface; thus, oxidation starts from edges [41]. However, the most challenging task for the development of MXenes with oxidation stability could be the defect passivation of MXenes using organic and/or inorganic ligands. Defect-passivated MXenes block the reactivity of water molecules and dissolved oxygen for the oxidation of MXene flakes; thus, the defect passivation of MXenes with organic/inorganic ligands is the most effective strategy for preventing oxidation.

**Fig. 7:**
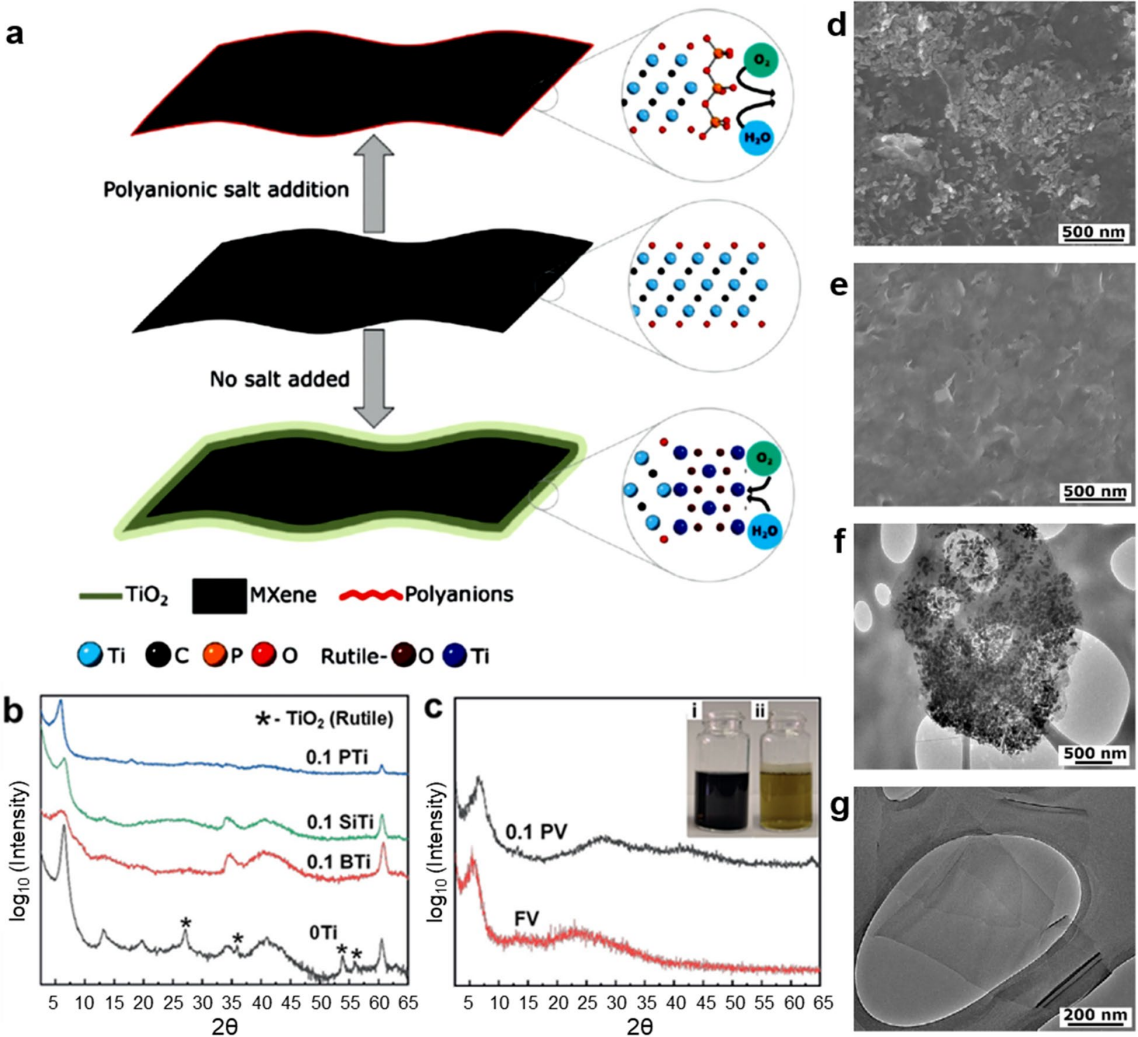
**a** Illustration for the edge capping of MXene sheets with polyanions. The middle flake represents a typical as-synthesized fresh MXene flake. The top flake shows that the edge-capping of MXene with polyanions protect it from oxidation, while the bottom bare flake undergoes oxidation over prolonged exposure to water and air. **b** XRD patterns of 0.1 M polyphosphate-Ti_3_C_2_T_*x*_ (blue), 0.1 M polysilicate-Ti_3_C_2_T_*x*_ (green), 0.1 M polyborate-Ti_3_C_2_T_*x*_ (red), and untreated Ti_3_C_2_T_*x*_ (black), **c** XRD patterns of 0.1 M polyphosphate-V_2_CT_*x*_ (black), and untreated-V_2_CT_*x*_ (red) samples. Patterns are shifted vertically for clarity. Inset shows digital photographs of (i) 0.1 M polyphosphate-V_2_CT_*x*_ (left) sample and (ii) untreated V_2_CT_*x*_ sample (right) after three weeks of exposure to ambient air. Peaks of rutile are marked by asterisks. Typical SEM images of **d** untreated Ti_3_C_2_T_*x*_ and **e** 0.1 M polyphosphate-Ti_3_C_2_T_*x*_ samples. Typical TEM images of **f** untreated Ti_3_C_2_T_*x*_ and **g** 0.1 M polyphosphate-Ti_3_C_2_T_*x*_ samples. **a**-**g** Reproduced with permissions from ref. [59]. Copyright (2019), Wiley–VCH

In 2019, Barsoum et al. [59] reported that edge-passivated Ti_3_C_2_T_x_ and V_2_CT_*x*_ MXenes with polyanionic salts improved the oxidation stability because of the edge capping effect. Figure 7a illustrates that pristine MXenes are vulnerable to the attacks by water molecules and dissolved oxygen and are easily oxidized and converted to TiO_2_. In contrast, the edge capping of MXenes with polyanions blocks the water molecules and dissolved oxygen, thereby improving the oxidation stability.

Since the edge of MXene is positively charged [60], the edges of Ti_3_C_2_T_*x*_ (Ti) and V_2_CT_*x*_ (V) MXenes are simply passivated with polyanionic salts, such as polyphosphates (P), polysilicates (Si), and polyborates (B). Figure 7b shows the XRD patterns of Ti_3_C_2_T_*x*_ (0Ti, black), polyphosphate-passivated Ti_3_C_2_T_*x*_ (0.1PTi, blue), polysilicate-passivated Ti_3_C_2_T_*x*_ (0.1SiTi, green), and polyborate-passivated Ti_3_C_2_T_*x*_ (0.1BTi, red) aqueous dispersions after three weeks of exposure to ambient air. The value before the sample name represents the molar concentration of polyanionic salts. The XRD results revealed that after three weeks of exposure to ambient air, the control Ti_3_C_2_T_*x*_ (Ti) sample without polyanions revealed the peaks of (002), (004), and (006) planes of MXene flakes at 6.5°, 13.0°, and 20.0°, respectively, as well as the clear peaks belonging to rutile TiO_2_, as indicated by asterisks (JCPDS-# 12–1276). This indicates that Ti_3_C_2_T_*x*_ (Ti) is partially oxidized to TiO_2_. These TiO_2_ peaks are absent in all other samples passivated with polyanions, even in those with a minimum salt concentration of 0.1 M, irrespective of their chemistry. This indicates that edge capping with polyanions prevents the oxidation of Ti_3_C_2_T_*x*_ MXene flakes during storage. Similarly, polyphosphate-passivated V_2_CT_*x*_ (0.1PV) showed a significant improvement in oxidation stability (Fig. 7c). The SEM and TEM analyses also confirmed that the polyphosphate-passivated Ti_3_C_2_T_*x*_ MXene sheets remained unoxidized, but the non-passivated MXene sheets were significantly oxidized to form TiO_2_ (Fig. 7d–g). This shows that oxidation stability of MXenes can be improved in a similar way, which remains equally effective irrespective of their different elemental and M_n_X_n+1_T_*x*_ compositions. This is attributed to the similar surface chemistry arising from same synthesis conditions.

**Fig. 8:**
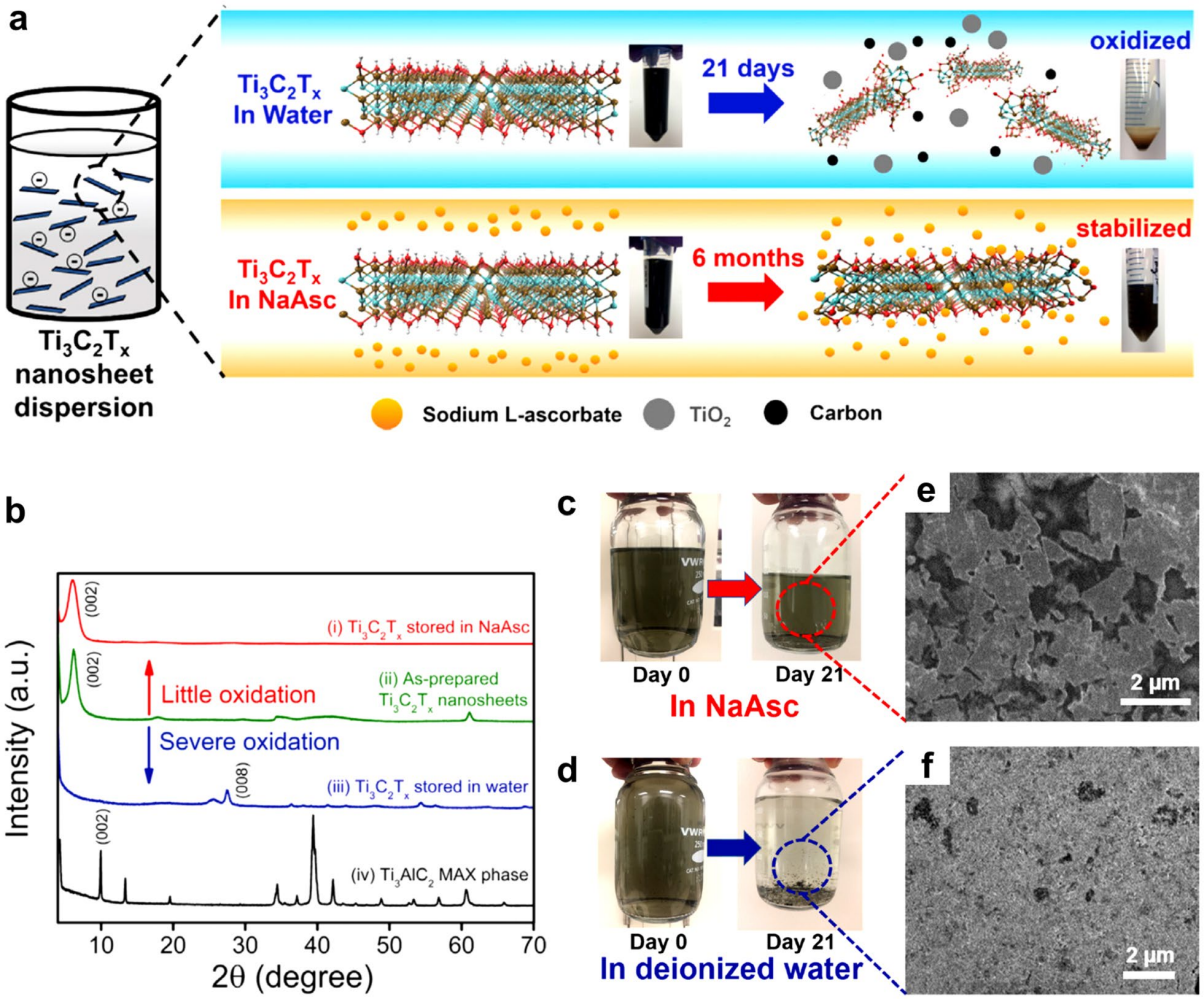
**a** Schematics of shelf-stable Ti_3_C_2_T_*x*_ nanosheet dispersion enabled by antioxidant sodium l-Ascorbate. **b** XRD patterns of Ti_3_AlC_2_ MAX phase, Ti_3_C_2_T_*x*_ stored in water for 21 days, as-prepared fresh Ti_3_C_2_T_*x*_, and Ti_3_C_2_T_*x*_ stored in NaAsc for 21 days. Digital photographs of the Ti_3_C_2_T_*x*_ suspensions on days 0 and 21 in **c** NaAsc and **d** deionized water. SEM images of Ti_3_C_2_T_*x*_ suspensions stored for 21 days in **e** NaAsc and **f** deionized water. **a**–**f** Reproduced with permissions from ref. [61]. Copyright (2019), Elsevier

**Fig. 9:**
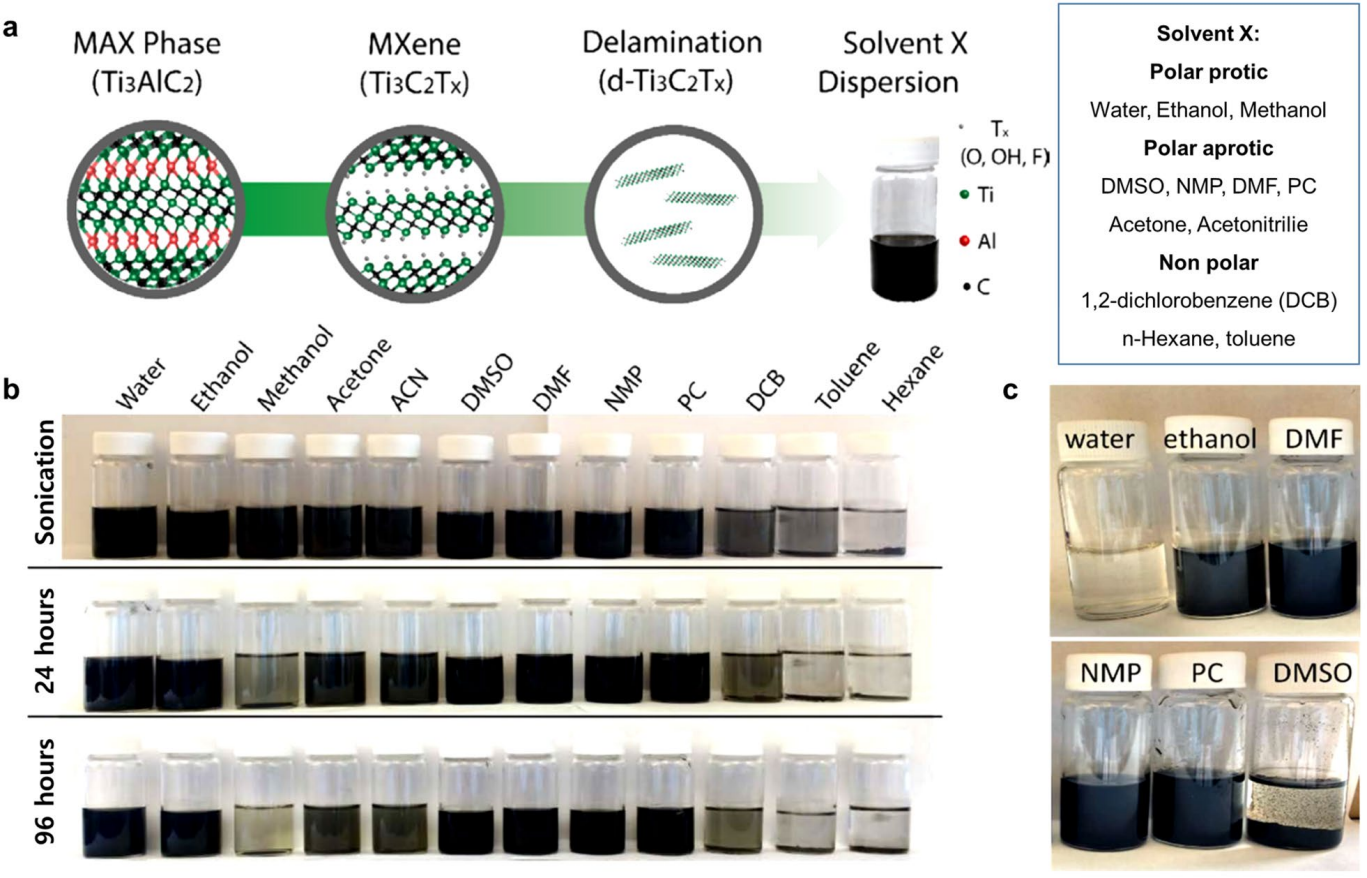
**a** Schematic describing the synthesis and dispersion of Ti_3_C_2_T_*x*_ in various organic solvents. **b** Dispersions of 50% HF-etched Ti_3_C_2_T_*x*_ in the 12 different solvents. Time points of just after sonication (top), 24 h after sonication (middle), and 96 h after sonication (bottom) were used to monitor stability. **c** Long-term dispersion stability (after 40 days) of Ti_3_C_2_T_*x*_ MXene in organic solvents and water. Ti_3_C_2_T_*x*_ in water oxidized (white sediment) while the dispersions in ethanol, DMF, NMP, PC stayed dark in color. Ti_3_C_2_T_*x*_ in DMSO agglomerated over time and formed a sediment but remained black (no visible oxidation). **a**–**c** Reproduced with permissions from ref. [39]. Copyright (2017), American Chemical Society

Green et al. [61] reported a similar surface passivation approach to Ti_3_C_2_T_*x*_ MXenes with sodium l-ascorbate (NaAsc) and citric acid. They suggested that NaAsc and citric acid act as antioxidants in aqueous Ti_3_C_2_T_*x*_ MXene suspensions and extend the shelf life of Ti_3_C_2_T_*x*_ suspensions (Fig. 8a). After being stored for three weeks, surface-passivated Ti_3_C_2_T_*x*_ with sodium l-ascorbate retained a distinct (002) peak at a 2*θ* angle of 6.5°, while the (002) peak of pristine MXene without any passivation completely disappeared from the XRD pattern, indicating that the crystalline Ti_3_C_2_T_*x*_ MXene was completely oxidized to TiO_2_ (Fig. 8b). These XRD results suggest that the introduction of NaAsc as an antioxidant prevents the oxidation of Ti_3_C_2_T_*x*_ MXene sheets. The Ti_3_C_2_T_*x*_ dispersions stored for 21 days in the NaAsc solution and water are compared in Fig. 8c and d, respectively. The color of the NaAsc-passivated Ti_3_C_2_T_*x*_ MXene solution did not change, but the untreated Ti_3_C_2_T_*x*_ flakes agglomerated, settled down, and oxidized. NaAsc-passivated Ti_3_C_2_T_x_ MXene retained its original sheet morphology (Fig. 8e); however, untreated Ti_3_C_2_T_*x*_ MXene was transformed into agglomerated TiO_2_ nanocrystals (Fig. 9f).

### Organic media or organic dispersions of MXenes

One of the most powerful methods to improve the oxidation stability of MXenes is to replace the water medium with an organic solvent. Aqueous MXene suspensions can be easily transformed into TiO_2_ via reactions with O_2_ and/or H_2_O molecules. The organic medium can exclude water and minimize contact with oxygen. Owing to the strong surface hydrophilicity of MXenes arising from the abundance of surface functional groups such as –OH, = O, and–F, pristine MXenes are well-dispersed in polar solvents, including dimethyl sulfoxide (DMSO), dimethylformamide (DMF), N-methyl-2-pyrrolidone (NMP), and propylene carbonate (PC). However, MXenes show poor dispersibility in other polar or nonpolar organic solvents. Therefore, dispersion in various organic solvents is quite challenging.

Yury and co-workers [39] reported the dispersion and oxidation stability of Ti_3_C_2_T_*x*_ MXene in various organic solvents (Fig. 9a, b). Pristine Ti_3_C_2_T_*x*_ MXene has excellent long-term dispersion stability (for 96 h) in organic solvents such as DMSO, DMF, NMP, PC, and ethanol (EtOH), but exhibits poor dispersion stability in other organic solvents. After being stored for 40 days, the aqueous Ti_3_C_2_T_*x*_ MXene suspensions were completely oxidized and transformed to colorless cloudy solutions, while Ti_3_C_2_T_*x*_ suspensions in ethanol, DMF, NMP, and PC were dark black (Fig. 9c). These results indicate that organic solvents prevent the oxidation of MXene flakes because the organic medium can exclude or minimize the water and/or dissolved oxygen molecules.

**Fig. 10:**
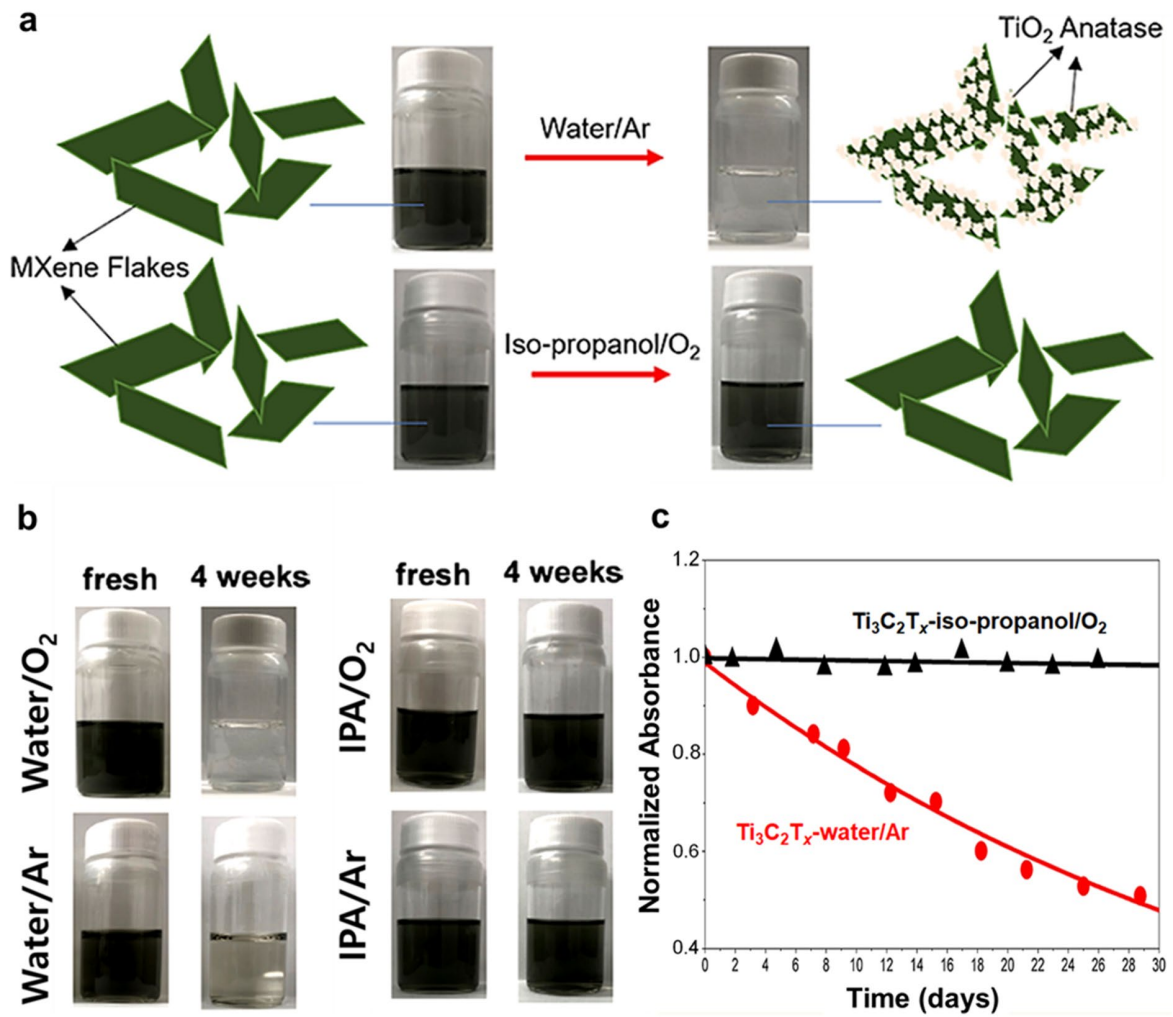
**a** Comparative schematic illustration of the oxidation stability of each newly prepared Ti_3_C_2_T_*x*_ MXene colloidal solution after purging with O_2_ or Ar. **b** Visual appearance of Ti_3_C_2_T_*x*_ MXene colloidal solutions in different environments over time. **c** Stability of Ti_3_C_2_T_*x*_ colloidal solutions in different environments over time. **a**–**c** Reproduced with permissions from ref. [38]. Copyright (2019), American Chemical Society

Huang and Mochalin [38] reported that Ti_3_C_2_T_*x*_ dispersions in isopropanol (IPA) were highly stable against oxidation under oxygen and argon (Ar) atmospheres, while Ti_3_C_2_T_*x*_ dispersions in water were unstable against oxidation under O_2_ and Ar (Fig. 10a–c). Aqueous Ti_3_C_2_T_*x*_ MXene dispersions became cloudy, whereas dispersions stored in IPA retained their original black color for both oxygen and Ar atmospheres (Fig. 10b). The UV–vis analysis of Ti_3_C_2_T_*x*_ MXene under water/Ar and IPA/O_2_ conditions was also conducted for verification (Fig. 10c). The normalized absorbance of the Ti_3_C_2_T_*x*_ peak at 760 nm in the UV–vis curve corresponded to the relative concentration of Ti_3_C_2_T_*x*_ flakes. The concentration of Ti_3_C_2_T_*x*_ MXenes stored in water/Ar decreased with time, indicating that oxidation progressed continuously; however, the concentration of Ti_3_C_2_T_*x*_ MXene in IPA/O_2_ did not change even after 30 days, indicating no oxidation. Based on a series of controlled experiments, it was concluded that the decomposition of aqueous Ti_3_C_2_T_*x*_ suspensions to TiO_2_ is due to hydrolysis by H_2_O, and not due to oxidation by O_2_ gas.

**Fig. 11:**
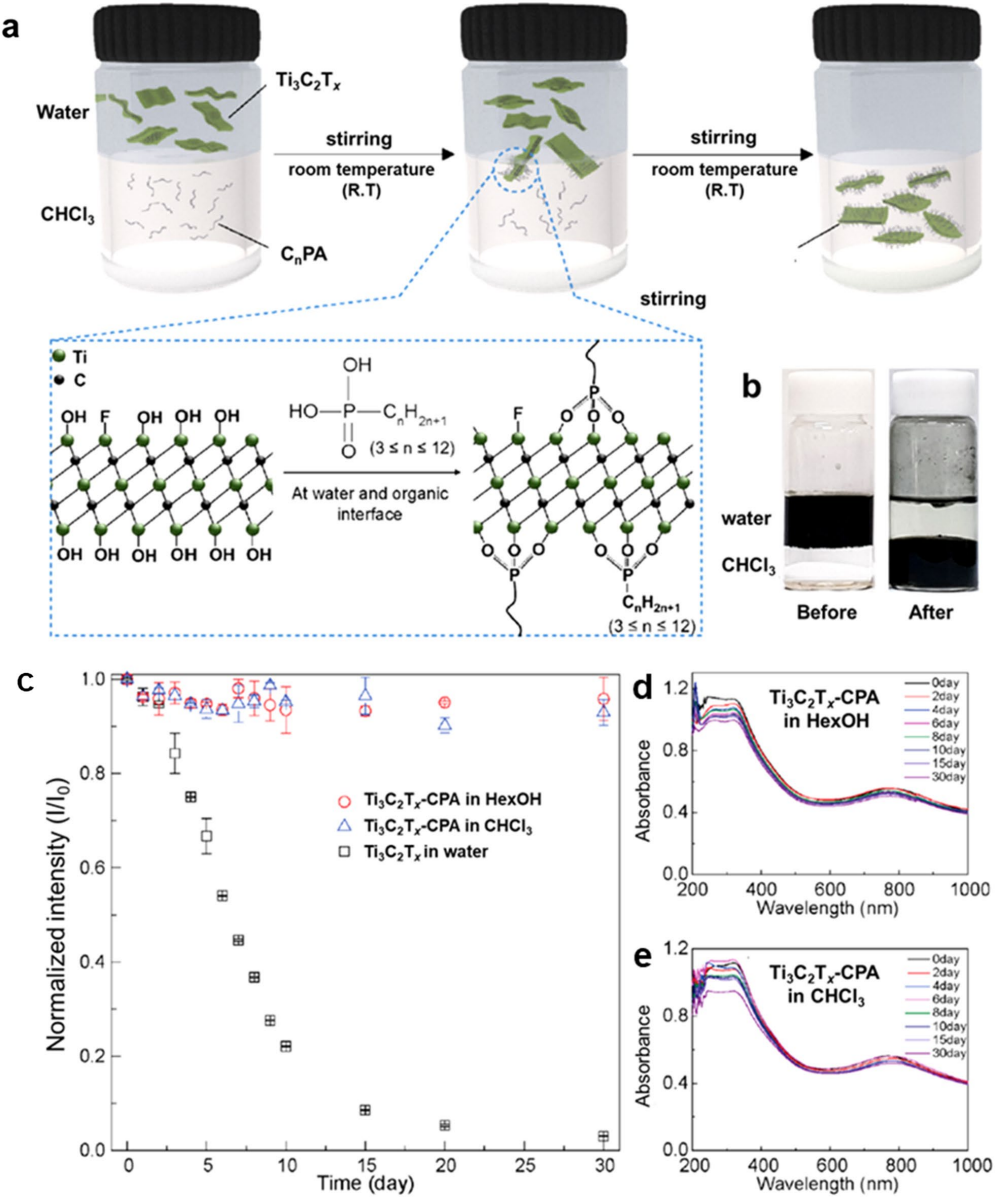
**a** Schematic illustration of simultaneous interfacial chemical grafting reaction and phase transfer for preparing a stable nonpolar Ti_3_C_2_T_*x*_ dispersion. **b** Photographs of Ti_3_C_2_T_*x*_ dispersion in immiscible water and chloroform phases before and after the reaction. **c** Normalized intensity of the peak at around 760 nm for pristine Ti_3_C_2_T_*x*_ in water, Ti_3_C_2_T_*x*_-C_12_PA in hexanol, and Ti_3_C_2_T_*x*_-CPA in chloroform as a function of storage time. UV − visible absorbance spectra of **d** Ti_3_C_2_T_*x*_-CPA in hexanol, and **e** Ti_3_C_2_T_*x*_-CPA in chloroform, respectively. **a**–**e** Reproduced with permissions from ref. [40]. Copyright (2019), American Chemical Society

Koo and co-workers (our group) [40] reported nonpolar organic dispersions of Ti_3_C_2_T_*x*_ MXene developed through simultaneous interfacial chemical grafting and phase transfer. Hydroxyl groups on the MXene flakes were chemically grafted with organic ligands to improve surface hydrophobicity. The Ti_3_C_2_T_*x*_ flakes were chemically grafted with alkyl phosphonic acids through interfacial nucleophilic addition and sequential condensation reactions (Fig. 11a); at the same time, these flakes migrated from the aqueous phase to the organic phase after reaction (Fig. 11b). Therefore, surface-functionalized MXenes became dispersible in nonpolar organic solvents, including hexanol and chloroform. The Ti_3_C_2_T_*x*_ MXene dispersions in nonpolar organic solvents revealed excellent oxidation stability. The aqueous pristine Ti_3_C_2_T_*x*_ suspensions were transformed to a translucent cloudy solution after 30 days due to complete oxidation, while alkyl phosphonic acid substituted Ti_3_C_2_T_*x*_ MXenes (Ti_3_C_2_T_*x*_-CPA) in chloroform or *n*-hexanol maintained the initial dark color after same storage time. The UV–vis absorption curves of Ti_3_C_2_T_*x*_ dispersions in hexanol, and chloroform and the change in the intensity of the absorbance peaks at 760 nm were monitored (Fig. 11c–e). The UV–vis results verified that the Ti_3_C_2_T_*x*_-CPA dispersions in both hexanol and chloroform organic solvents are highly stable against chemical oxidation.

**Fig. 12:**
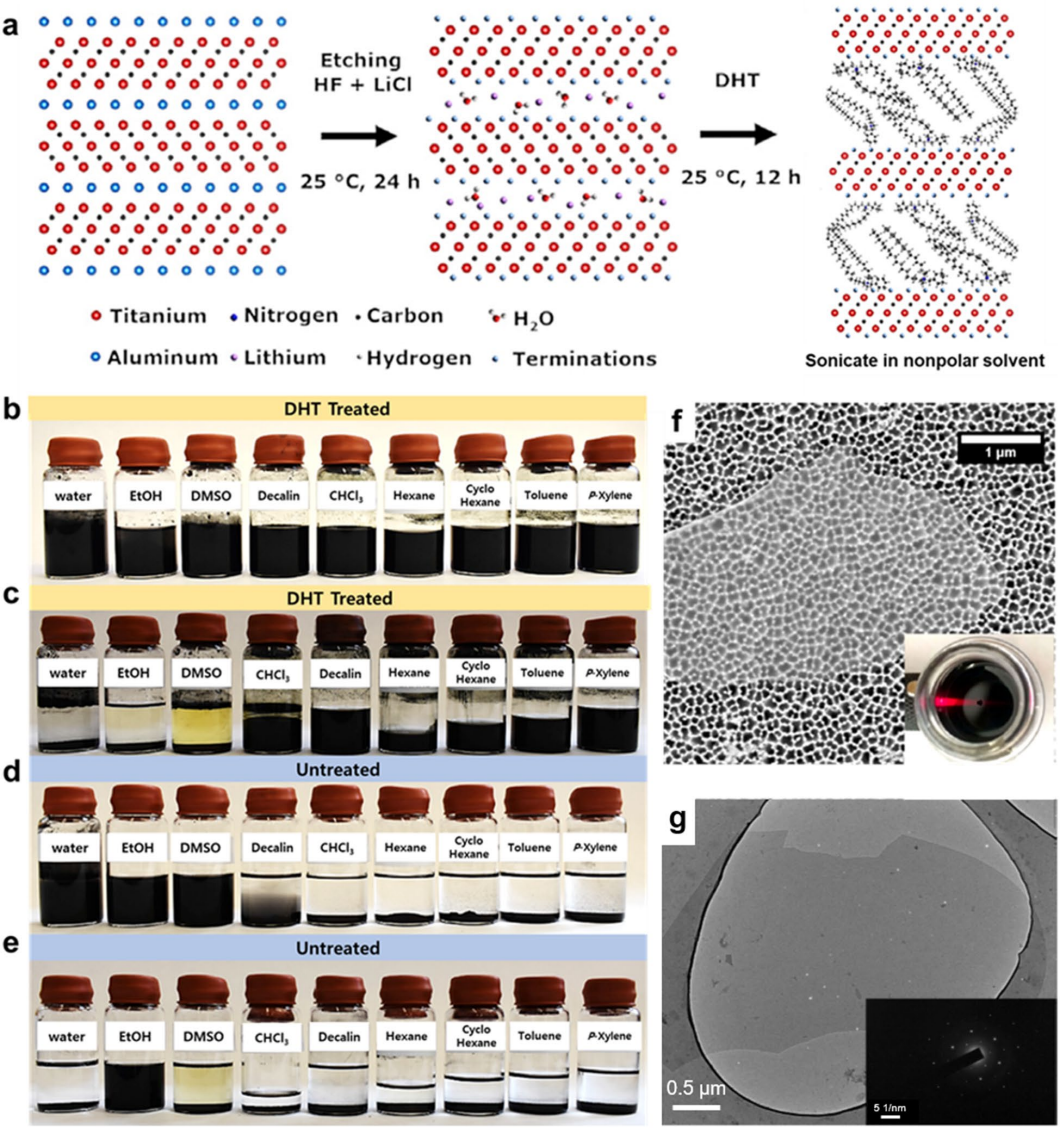
**a** Schematic illustration of simultaneous interfacial chemical grafting reaction and phase transfer for preparing a stable nonpolar Ti_3_C_2_T_*x*_ dispersion. Photographs of DHT-treated Ti_3_C_2_T_*x*_ suspensions in polar (first three vials) and nonpolar solvents **b** right after sonication and **c** after 10 days. Photographs of Ti_3_C_2_T_*x*_ suspensions **d** right after sonication and **e** after 10 days. **f** Typical SEM micrograph of a drop of DHT-treated Ti_3_C_2_T_*x*_ dispersed in a cyclohexane suspension cast on a porous alumina substrate. Scale bar is 1 μm. Inset shows the Tyndall effect. **g** TEM micrograph of a drop of a DHT-MXene-decalin suspension after 10 days. Scale bar is 0.5 μm. Inset shows selected area diffraction pattern with a scale bar of 10 nm^−1^. **a**–**g** Reproduced with permissions from ref. [62]. Copyright (2020), Elsevier

Barsoum and co-workers [62] reported another nonpolar solvent dispersion of Ti_3_C_2_T_*x*_ MXenes functionalized with di(hydrogenated tallow)benzyl methyl ammonium chloride (DHT). The Li^+^ ions present in the multilayer space were exchanged for DHT (Fig. 12a), and the DHT-functionalized Ti_3_C_2_T_*x*_ MXene became organophilic and dispersible in nonpolar solvents such as decalin, chloroform, hexane, and toluene (Fig. 12b–c). In contrast, untreated Ti_3_C_2_T_*x*_ MXenes were hydrophilic and dispersible in polar solvents such as water, EtOH, and DMSO (Fig. 12d–e). SEM and TEM analyses confirmed that the DHT-substituted Ti_3_C_2_T_*x*_ MXene sheets were not oxidized; they maintained a clean and original sheet shape even after stored for 10 days (Fig. 12f, g).

### Polymeric composites of MXenes

Preparing MXene-polymer composites is a promising method to not only improve the oxidation stability of MXenes, but also to enhance the mechanical strength of the MXene films; this is because a flexible polymer generally exhibits high mechanical strength and can isolate MXene flakes from oxygen and water [12, 14, 63, 64].

**Fig. 13:**
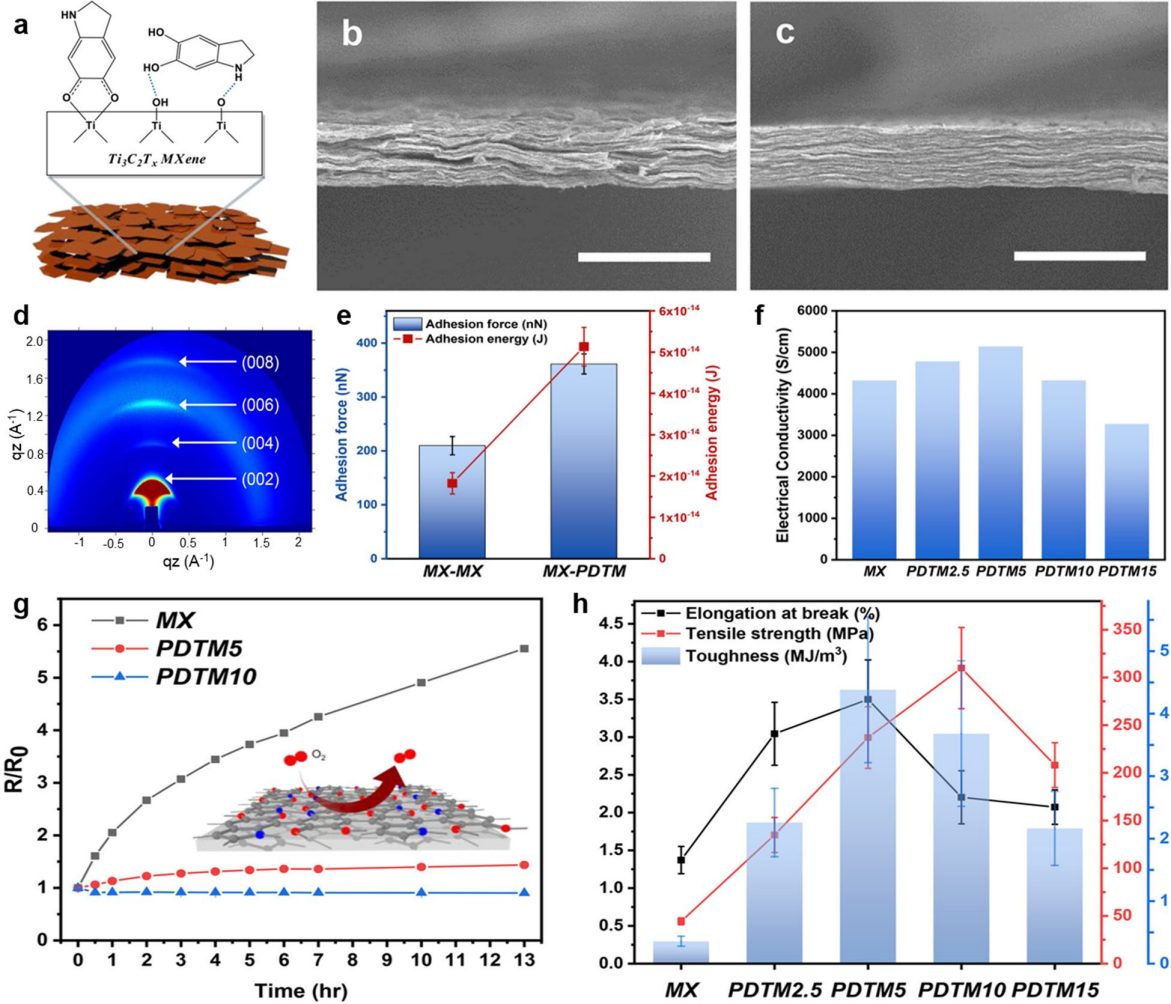
**a** Schematic illustration of fabrication procedure polydopamine-treated MXene composite (PDTM). SEM cross-sectional images of **b** MXene and **c** PDTM5 (scale bar: 20 μm). **d** GIWAXS patterns of PDTM5. **e** Adhesion force and energy calculated from the force-distance curve. **f** Electrical conductivity of each sample. **g** Electrical resistance change of pure MXene and polydopamine-treated MXene composites upon heating (170 °C) in air. **h** Calculated tensile strength, elongation, and toughness of neat MXene and its PDTM composites. **a**–**h** Reproduced with permissions from ref. [65]. Copyright (2020), American Chemical Society

Lee et al. [65] reported that the oxidation stability of MXene flakes can be improved by preparing MXene-polydopamine composites. Dopamine monomers were polymerized in-situ to polydopamine on the surface of Ti_3_C_2_T_*x*_ (MXene) flakes without an initiator by spontaneous interfacial charge transfer of dopamine (Fig. 13a). The compact MXene/polydopamine composite film was prepared using vacuum filtration, revealing a better orientation order than that of the neat MXene film (Fig. 13b–d), owing to the strong adhesion arising from the polydopamine coated on the surface of MXenes (Fig. 13e). It caused the MXene-polydopamine composite with 5 wt% polydopamine (PDTM5) to exhibit a higher electrical conductivity than that of the pristine MXene film (Fig. 13f**)**. Figure 13g shows the change in electrical resistance of neat MXene and MXene-polydopamine composite films as a function of annealing time at 170 °C in air. Pure MXene films showed an increase of five-fold or more in resistance after 13 h. PDTM5 composite film showed a smaller increase in resistance, whereas PDTM10 with 10wt% polydopamine showed a negligible increase in the resistance under the same annealing conditions. These results indicate that the introduction of polydopamine to the composites improves the oxidation stability of MXene flakes, and 10 wt% loading of polydopamine can even reduce the resistance due to the shrinkage of the interlayer gap at elevated temperatures [13]. XPS depth profiles revealed that even after an hour of heat treatment at 170 °C, oxidation occurred mostly on the surface areas, and not in the middle layer of the PDTM5 film. This suggests that the MXene dopamine composite is densely packed due to the binding between catechol and amine groups of dopamines and the hydroxyl groups of MXene flakes, which can prevent the penetration of oxygen and humidity. Furthermore, the introduction of polydopamine improved the tensile strength by 5.35 times (237.0 MPa) and 6.99 times (309.8 MPa) for PDTM5 and PDTM10, respectively, compared to that of the pure MXene film (Fig. 13h).

## Summary and perspectives

**Fig. 14:**
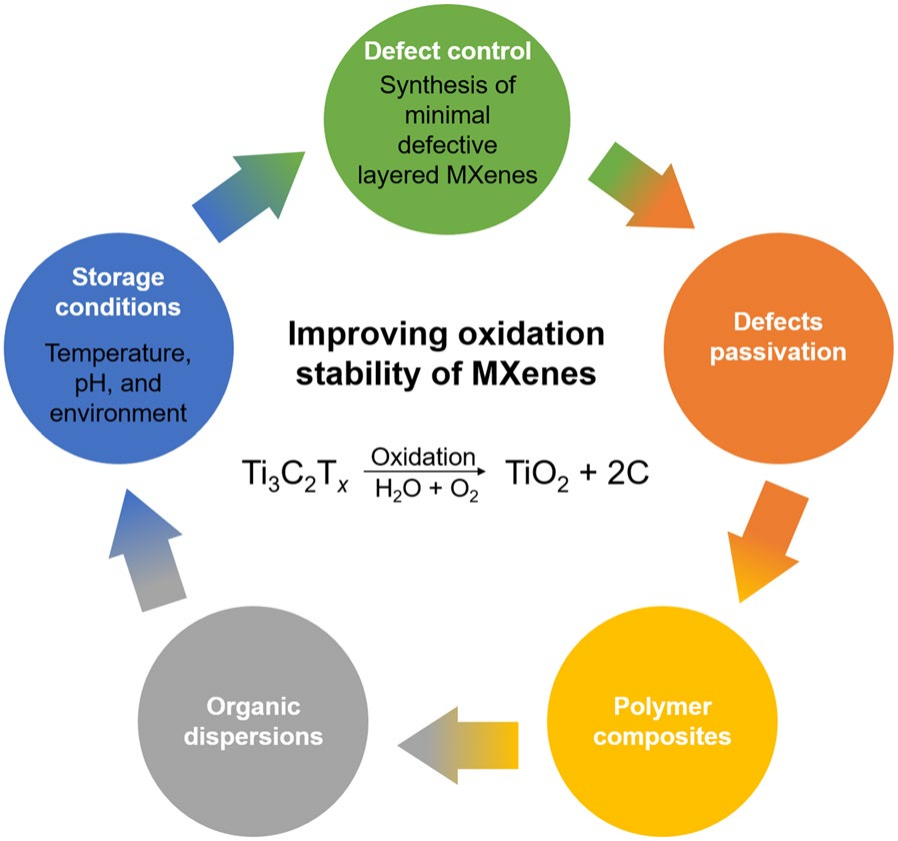
Control factors for preventing oxidation of MXenes

This study highlights the importance of improving the oxidation stability of highly conductive multifunctional 2D MXenes and comprehensively reviews available research on how to control their oxidation kinetics. The defective sites at the edge or on the surface of MXene flakes, which originate from the harsh chemical etching synthesis, act as active sites for the oxidative degradation reaction with water and/or oxygen and cause high crystalline transition metal carbide/carbonitride MXenes to transform into transition metal oxides such as TiO_2_ and amorphous carbon. Oxidative degradation deteriorates the intrinsic properties of MXenes. This oxidative decomposition of MXenes can be significantly delayed by controlling various pre-synthesis and post-synthesis parameters including the quality of the parent MAX phase, chemical etching conditions such as the type and concentration of acid etchants, ultrasonication, and storage environment such as storage media, temperature, pH, and the concentration of the aqueous dispersions (see the summarized Fig. 14). First of all, type and quality of the starting materials for the synthesis of MAX and MXene directly affect the properties of the ensuing MXene. A high-quality MAX phase synthesized by pressureless sintering will ensure a good-quality MXene dispersion after etching in mild acidic conditions, which will favor an easy washing of the product, and induce fewer atomic defects which, subsequently, will guarantee a prolonged shelf life of their aqueous dispersions. Selection of a minimal intensive layer delamination process will produce MXenes with larger flake size which owe a higher oxidation stability than smaller flakes, due to the decreased density of defective sites such as defects and edges. A proper synthesis process can help eliminating the step of ultrasonication which shatters MXene flakes and reduces their average lateral size. After careful synthesis of a good-quality MXene, its storage is another challenging task to use it for a longer period of time with maximum reproducibility of the results. For this purpose, an aqueous dispersion of MXene of neutral pH can be stored for couple of months if stored in a lower temperature and inert atmosphere. As dissolved oxygen and water are the main oxidation sources, removal of oxygen and water is favorable. Extending this approach, aqueous dispersions of MXenes can be replaced with their dispersions in organic solvents, minimizing the exposure to water and oxygen molecules, hence extending their oxidation stability. Recent studies revealed an edge capping strategy with negatively charged ligands to lower the oxidation behavior of MXenes as defective edge sites of their flakes are believed to be positively charged. We believe that non-aqueous etching of MAX phases to synthesize MXenes, with more improved quality than the few reported studies, will have a high impact on all these challenges in the stability of MXenes. Introduction of conventional polymers can reduce the degree of oxidation of MXenes, simultaneously improving their mechanical properties for real applications. In other ways, thin MXene films can be sandwiched between polymer coatings, fulfilling the needs of flexible electronics with improved oxidation resistance along with superb mechanical strength. Despite promising experimental advancements, the mechanism of the oxidative degradation reaction of aqueous Ti_3_C_2_T_*x*_ MXene dispersions is not yet fully understood. Completely preventing the oxidation of Ti_3_C_2_T_*x*_ and other MXenes is also quite challenging, especially when they are transported from laboratories to industries for developing commercial products. Therefore, MXenes have prompted the scientific community to investigate their oxidation kinetics and mechanisms and develop new strategies to improve their oxidation stability.

## Data Availability

Not applicable.

## Abbreviations

2D: Two-dimensional
LEDs: Light-emitting diodes
3D: Three-dimensional
HF: Hydrofluoric
EMI: Electromagnetic interference
BP: Black phosphorous
HAADF: High-angle annular dark field
STEM: Scanning transmission electron microscopy
TEM: Transmission electron microscopy
FFT: Fast Fourier transform
EELS: Electron energy loss spectroscopy
LiF: Lithium fluoride
HCl: Hydrochloric
MILD: Minimal intensive layer delamination
XRD: X-ray diffraction
SEM: Scanning electron microscopy
XPS: X-ray photoelectron spectroscopy
PVA: Polyvinyl alcohol
NaAsc: Sodium l-ascorbate
IPA: Isopropanol
DMSO: Dimethyl sulfoxide
DMF: Dimethylformamide
NMP: *N*-Methyl-2-pyrrolidone
PC: Propylene carbonate
EtOH: Ethanol
DHT: Di(hydrogenated tallow)benzyl methyl ammonium chloride
PDTM: 5MXene-polydopamine composite
